# Antibiotic-induced cellular enlargement drives phage synergy and resensitizes carbapenem-resistant *Acinetobacter baumannii*

**DOI:** 10.1128/spectrum.01424-26

**Published:** 2026-08-28

**Authors:** Arrita Rueangrit, Wanna Sudhikaran, Arom Sombutmark, Pisamai Pinsrithong, Lalitwadee Chotuk, Ampapan Naknaen

**Affiliations:** 1 Department of Biomedical Sciences and Biomedical Engineering, Faculty of Medicine, Prince of Songkla University 37689, Hat Yai, Songkhla, Thailand; 2 Department of Pathology, Faculty of Medicine, Prince of Songkla University 37689, Hat Yai, Songkhla, Thailand; 3 Office of Scientific Instrument and Testing, Prince of Songkla University 26686, Hat Yai, Songkhla, Thailand; 4 Department of Ophthalmology, Faculty of Medicine, Prince of Songkla University 37689, Hat Yai, Songkhla, Thailand; Rowan University Cooper Medical School, Camden, New Jersey, USA

**Keywords:** carbapenem-resistant *Acinetobacter baumannii*, phage-antibiotic synergy, phage therapy, antibiotic resensitization, bacterial morphology change

## Abstract

Carbapenem-resistant *Acinetobacter baumannii* (CRAB) represents a critical clinical challenge, as phage monotherapy is frequently compromised by the rapid emergence of bacterial resistance. This study characterizes phiAR008, a newly isolated *Hadassahvirus* phage with therapeutic potential against clinical CRAB isolates, and evaluates its synergistic efficacy alongside five antibiotic classes to develop robust combination therapies. Although phiAR008 monotherapy initially reduced bacterial growth, rapid bacterial regrowth occurred. In contrast, combination treatment with subinhibitory concentrations of meropenem (MEM) or ciprofloxacin (CIP) produced complete bacterial clearance *in vitro* and significantly enhanced antibacterial activity compared with either treatment alone. MEM and CIP induced distinct morphological changes, including spheroid enlargement and filamentation, respectively, resulting in increased bacterial cell surface area. These morphological alterations were associated with accelerated phage adsorption and increased intracellular phage accumulation during the early stages of infection, whereas the final phage yield remained unchanged, suggesting that antibiotic-induced cell enlargement primarily enhances early infection kinetics rather than phage productivity. In contrast, antibiotics that did not substantially alter bacterial morphology, including colistin, rifampin, and chloramphenicol, exhibited limited synergy with phiAR008. In a *Galleria mellonella* infection model, phiAR008-MEM and phiAR008-CIP combination therapy significantly improved larval survival compared with phage or antibiotic monotherapy. Phage-resistant isolates recovered following combination treatment also exhibited reduced virulence and altered antibiotic susceptibility. Although the genetic and mechanistic basis of these phenotypic changes remains to be determined, our findings indicate that antibiotic-induced bacterial morphological changes may contribute to phage-antibiotic synergy by enhancing early phage-host interactions. These results support the rational optimization of phage-antibiotic combinations as a promising therapeutic strategy for refractory CRAB infections.

**IMPORTANCE:**

Phage-antibiotic synergy offers a promising strategy to overcome the therapeutic limitations of phage monotherapy against CRAB. In this study, phiAR008 showed limited standalone killing because bacterial regrowth and phage resistance emerged rapidly. However, when combined with specific antibiotics, especially meropenem and ciprofloxacin, phiAR008 produced strong synergistic antibacterial activity, leading to rapid and complete bacterial eradication *in vitro* and improved survival *in vivo*. Importantly, synergy was not universal across antibiotic classes, indicating that the choice of partner drug is critical. Our findings further show that synergy is mechanistically linked to antibiotic-induced changes in bacterial morphology that enhance phage adsorption rather than phage production. These results provide practical guidance for selecting phage-antibiotic pairs and support phage-antibiotic synergy as a mechanistically informed approach for treating multidrug-resistant *A. baumannii* infections.

## INTRODUCTION

*Acinetobacter baumannii* is a formidable nosocomial pathogen responsible for severe, life-threatening infections among critically ill patients (1, 2). Its exceptional environmental resilience and propensity for clonal spread drive persistent hospital outbreaks (1, 3). Compounding this threat is the pathogen’s vast repertoire of intrinsic and acquired antimicrobial resistance (4). Most critically, the global proliferation of carbapenem-resistant *A. baumannii* (CRAB) has systematically depleted frontline therapeutic options, elevating it to a top-tier public health crisis (3). While multidrug-resistant international clones dominate globally, the emergence of distinct local lineages further complicates clinical management and underscores the urgent need for novel therapeutic strategies (5). As traditional antibiotic pipelines struggle to keep pace with the rapid evolution of multidrug-resistant CRAB, the development of alternative therapeutic interventions has become an urgent clinical priority.

Therapeutically, strictly lytic bacteriophages (phages) are preferred over temperate phages to avoid the unintended lysogenic transfer of virulence or resistance genes, relying instead on rapid, holin-endolysin-mediated cell lysis (6–8). Leveraging these unique, resistance-circumventing mechanisms, phage therapy is undergoing a major clinical renaissance as a highly targeted alternative to conventional antibiotics. Accumulating preclinical data and successful global compassionate-use cases underscore the efficacy of lytic phages against multidrug-resistant bacteria in clinical scenarios where frontline treatments fail (9). However, despite this profound potential, the widespread translation of phage monotherapy is critically hindered by the rapid evolution of multifaceted bacterial resistance. In *A. baumannii*, the efficacy of standalone therapy is severely undermined by structural surface adaptations, specifically profound capsular phase variability and mutations of alternative, non-capsular receptors that effectively block phage adsorption (10, 11). Additionally, the bacteria utilize robust biofilm formation to physically shield itself from viral penetration (12), whereas classical CRISPR-Cas adaptive immunity plays only a marginal role in resistant nosocomial lineages (13). Because *A. baumannii* readily alters multiple surface targets to evade infection, this swift adaptation frequently precipitates pathogen regrowth and clinical relapse. Consequently, monotherapies are fundamentally insufficient for complete bacterial eradication, underscoring an urgent clinical need for synergistic, combinatorial therapeutic strategies.

To overcome these limitations, phage-antibiotic combination therapy has emerged as a highly promising clinical paradigm. By engaging in phage-antibiotic synergy (PAS), these regimens consistently deliver superior antibacterial activity against *A. baumannii* and suppress resistance evolution far more effectively than standalone treatments (14–18). Clinically, PAS capitalizes on complementary dynamics to lower the required antibiotic doses, eradicate recalcitrant biofilms, and actively exploit evolutionary trade-offs. As bacteria mutate to evade phages, they frequently incur fitness costs that attenuate their virulence or restore susceptibility to traditional antibiotics (18–21). Beyond these evolutionary pressures, PAS is driven by profound physiological interactions. Sublethal exposure to specific antibiotics can fundamentally alter bacterial cell surface architecture, notably inducing cellular enlargement or filamentation (22–25). However, the precise mechanistic link between these morphological shifts and enhanced viral kinetics remains a subject of intense debate. The prevailing “delayed lysis hypothesis” postulates that antibiotic-induced cellular expansion dilutes phage holin proteins, thereby prolonging the latent period to ultimately yield a larger burst size per cell (26, 27). Conversely, conflicting evidence demonstrates that PAS can actually accelerate lysis while still achieving high virion yields at the population level (28–30). While historical models attributed synergy almost exclusively to increased burst size (27, 28), recent findings indicate that antibiotic-induced morphological changes themselves, such as filamentation or cell bloating, are the physical drivers that accelerate phage propagation and expand lysis plaques (24, 25, 27, 28, 31). This mechanistic complexity underscores the critical need for comprehensive models that integrate both viral kinetics and antibiotic-altered host morphology to fully optimize combinatorial therapies for clinical use.

Here, to elucidate the mechanistic drivers of structural PAS, this study characterizes a newly isolated *Hadassahvirus* phage, phiAR008, which exhibits infectivity against several clinical CRAB strains but lacks sustained bactericidal efficacy as a monotherapy due to the rapid bacterial regrowth. We systematically evaluated the synergistic potential of phiAR008 in combination with diverse antibiotic classes to determine whether specific mechanisms of action, namely the disruption of cell wall synthesis (meropenem; MEM) or DNA replication (ciprofloxacin; CIP), trigger morphological shifts that accelerate viral kinetics. Using *in vitro* kinetic assays and an *in vivo Galleria mellonella* infection model, we hypothesized that antibiotic-induced cellular enlargement primarily drives synergy by physically expanding the cell surface area, thereby enhancing initial phage adsorption and accelerating bacterial clearance. Furthermore, we investigated the evolutionary consequences of this combined selection pressure to evaluate its potential to drive therapeutic trade-offs, such as the resensitization of emergent phage-resistant mutants to conventional antibiotics.

## RESULTS

### Phage phiAR008 morphological, biological, and genomic properties

**Fig 1:**
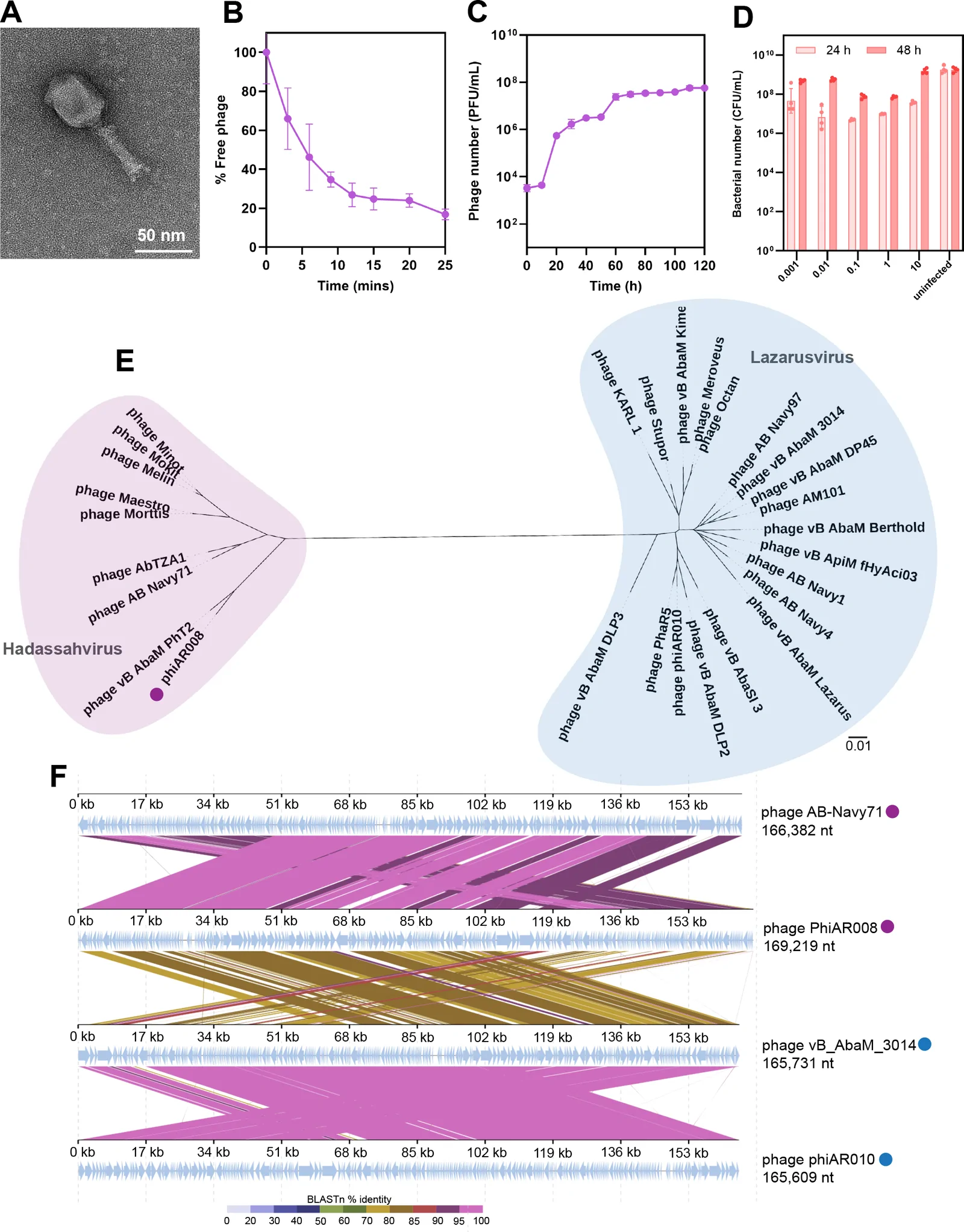
Morphological, biological, and genomic characterization of phage phiAR008. (**A**) Transmission electron micrograph of phiAR008 (scale bar, 50 nm). (**B**) Adsorption assay of phiAR008 on CRAB strain PT061 over 25 min. (**C**) One-step growth curve of phiAR008 in CRAB strain PT061 over 120 min. (**D**) Survival of the CRAB PT061 in the presence of phiAR008 at different MOIs (0.001, 0.01, 0.1, 1, and 10) after 12 and 24 h, indicating limited killing efficiency and increasing phage resistance over time. (**E**) Phylogenetic analysis based on whole-genome comparisons of selected *Hadassahvirus* and *Lazarusvirus* phages, placing phiAR008 within the *Hadassahvirus* cluster; the tree was generated using GBDP via VICTOR and visualized with iTOL. (**F**) Comparative genomic analysis of phiAR008 and representative *Hadassahvirus* phages; blue arrows indicate coding sequences, and shaded regions indicate sequence homology. Data in panels **B–D** are presented as mean ± SD of at least three independent experiments.

Phage phiAR008 was successfully isolated from influent wastewater collected from the student dormitories at Prince of Songkla University, Hat Yai Campus, Songkhla, Thailand, using the CRAB strain PT061 as the host bacterium. The phage produced clear plaques with an average diameter of 1.5 ± 0.53 mm (*n* = 35) on CRAB lawns (Fig. S1A), indicating strong lytic activity against the bacterial host. Transmission electron microscopy revealed that phiAR008 belongs to the class *Caudoviricetes* and exhibits morphological characteristics consistent with myoviruses, which are typically defined by icosahedral heads and contractile tails (32). The phage phiAR008 exhibited capsids measuring 76 ± 5 nm in width and 113 nm ± 5 in length, along with contractile tails measuring 129 ± 2 nm in length and 23 ± 1 nm in width (*n* = 3) (Fig. 1A). Regarding phage adsorption ability, the majority of phiAR008 (~80%) rapidly attached to the host cells and completed its replication cycle within approximately 10 min (Fig. 1B and C). The burst size was estimated at around 245 virions released per infected bacterial cell. To further evaluate their potential for medical applications, the host range was assessed against 18 clinical CRAB isolates obtained from Songklanagarind and Phatthalung Hospitals (33). The results showed that most of the clinical isolates were susceptible to the phage (11/18), whereas the phage did not infect *A. baumannii* ATCC 19606, *Staphylococcus aureus*, *Pseudomonas aeruginosa*, *Klebsiella pneumoniae*, and *Escherichia coli* (Table 1). We then evaluated phage infectivity by determining the efficiency of plating (EOP). Among all strains tested, only one strain (PT003) exhibited an EOP comparable to that of the parental host, PT061, whereas the remaining susceptible strains showed lower EOP values, indicating reduced plating efficiency. Nevertheless, the phage was able to infect the majority of clinical *A. baumannii* isolates, highlighting its potential for therapeutic application in clinical settings. Because phiAR008 exhibited favorable characteristics as a potential agent for bacterial growth control, an *in vitro* killing assay was conducted at 24 and 48 h to evaluate its lytic activity against CRAB at different multiplicities of infection (MOIs). However, phiAR008 showed only limited antibacterial efficacy, even at the highest MOI of 10 (Fig. 1D). At 24 h, CRAB counts were reduced by approximately 1–2 log CFU/mL relative to untreated control. By 48 h, bacterial regrowth was evident at all MOIs and was comparable to that of bacteria alone, suggesting the possible emergence of phage-resistant bacterial populations over time.

**TABLE 1:** Bactericidal spectrum and efficiency of plating (EOP) of the phage phiAR008^*a*^

Bacterial types	Strain code	Isolation sources	EOP value
*Acinetobacter baumannii*	PT061	Clinical isolates from Phatthalung Hospital, Thailand	1.00
	PT054		≤0.001
	PT058		≤0.001
	PT003		0.949
	PT009		≤0.001
	PT022		≤0.001
	PT071		–
	PT073		–
	PT005		–
	PSU051	Clinical isolates from Songklanagarind Hospital, Thailand	≤0.001
	PSU052		–
	PSU116		–
	PSU068		≤0.001
	PSU100		–
	PSU131		≤0.001
	PSU078		≤0.001
	PSU139		–
	PSU104		≤0.001
	DMST 2071	Department of Medical Sciences, Ministry of Public Health, Thailand	–
	ATCC19606	American Type Culture Collection	–
*Staphylococcus aureus*	ATCC29312		–
*Pseudomonas aeruginosa*	ATCC 27853		–
*Klebsiella pneumoniae*	ATCC700603		–
*Escherichia coli*	ATCC25922		–

^*a*^ EOP values were classified as highly productive (≥0.5), medium productive (0.1 ≤ EOP < 0.5), low productive (0.001 < EOP < 0.1) or inefficent (≤0.001). The “–” symbol indicates no infection.

The genome of phiAR008 is presented as a representative for this group of *Acinetobacter* myophages (Table S1). Phage phiAR008 contains a double-stranded DNA genome of 169,219 bp and a GC content of 36.6%. Eight tRNA genes were identified as Trp, Ser, Pro, Ser, Arg, Thr, Met, and Pyl with their anticodon sequences CCA, GCT, TGG, TGA, TCT, TGT, CAT, and CTA, respectively (Table S2). Phage phiAR008 encodes 260 open reading frames (ORFs), approximately 74% of which are annotated as hypothetical proteins with unknown functions. No genes encoding phage integrases or bacterial virulence-associated proteins were detected. A conserved core of 52 genes encoding proteins with direct identity to those of *Acinetobacter* phages (BLASTp, E < 10^−5^) was identified in several regions of the genome (Table S1). These genes include those encoding structural proteins as well as proteins involved in DNA replication and nucleotide metabolism. The capsid proteins, tail proteins, baseplate hub subunit proteins, tail fiber proteins, and prohead core proteins involved in structure and morphogenesis were identified, along with genes associated with DNA replication and transcription, including dCMP deaminase, exonuclease, DNA helicases, endonuclease VII, transcriptional regulators, and RNA ligase (Table S1). The endolysin lysis gene in phiAR008 (*gp176*) is arranged similarly to those of *Acinetobacter* phage AbTZA1 and is classified within glycoside hydrolase family 24 (34). This suggests a mechanism involving destabilization of the bacterial outer membrane, thereby facilitating access of the catalytic domain to the peptidoglycan layer and ultimately leading to cell lysis. Moreover, phage phiAR008 encodes a lysis inhibition protein (*gp122*), which is homologous to that of *Acinetobacter* phage vB_AbaM_PhT2 (35) and is likely involved in lysis inhibition (36). Although the effects of lysis inhibition in therapeutic interventions remain unclear, superinfection-induced lysis inhibition delays lysis timing and increases burst size *in vitro*, which may in turn influence phage proliferation *in vivo* at the site of therapeutic application (20).

Next, we examined the relatedness of phiAR008 to other known *Acinetobacter* phages and found that it shared high sequence similarity (99%) with phage AB-Navy71 (37), which is classified within the genus *Hadassahvirus*, based on MegaBLAST analysis. Intergenomic similarity analysis using the Virus Intergenomic Distance Calculator (VIRIDIC) demonstrated that phiAR008 shares 96% similarity with AB-Navy71, exceeding the 95% threshold commonly applied for species demarcation and supporting its classification within the genus *Hadassahvirus* (Fig. S1B). Phylogenomic analysis based on the VICTOR Genome-BLAST Distance Phylogeny (GBDP) platform further positioned phiAR008 within a cluster of closely related *Acinetobacter* phages belonging to the genus *Hadassahvirus*, clearly separated from members of the *Lazarusvirus* genus, as illustrated in Fig. 1E. Consistent with the phylogenetic tree, phiAR008 showed high similarity to *Acinetobacter* phage AB-Navy71. Comparative genomic analysis between phiAR008 and AB-Navy71 highlighted a high degree of similarity while also revealing distinct differences when compared to the more distantly related *Lazarusvirus* phages vB_AbaM_3014 and phiAR010 (Fig. 1F).

### Phage-antibiotic synergy results from unique combination

**Fig 2:**
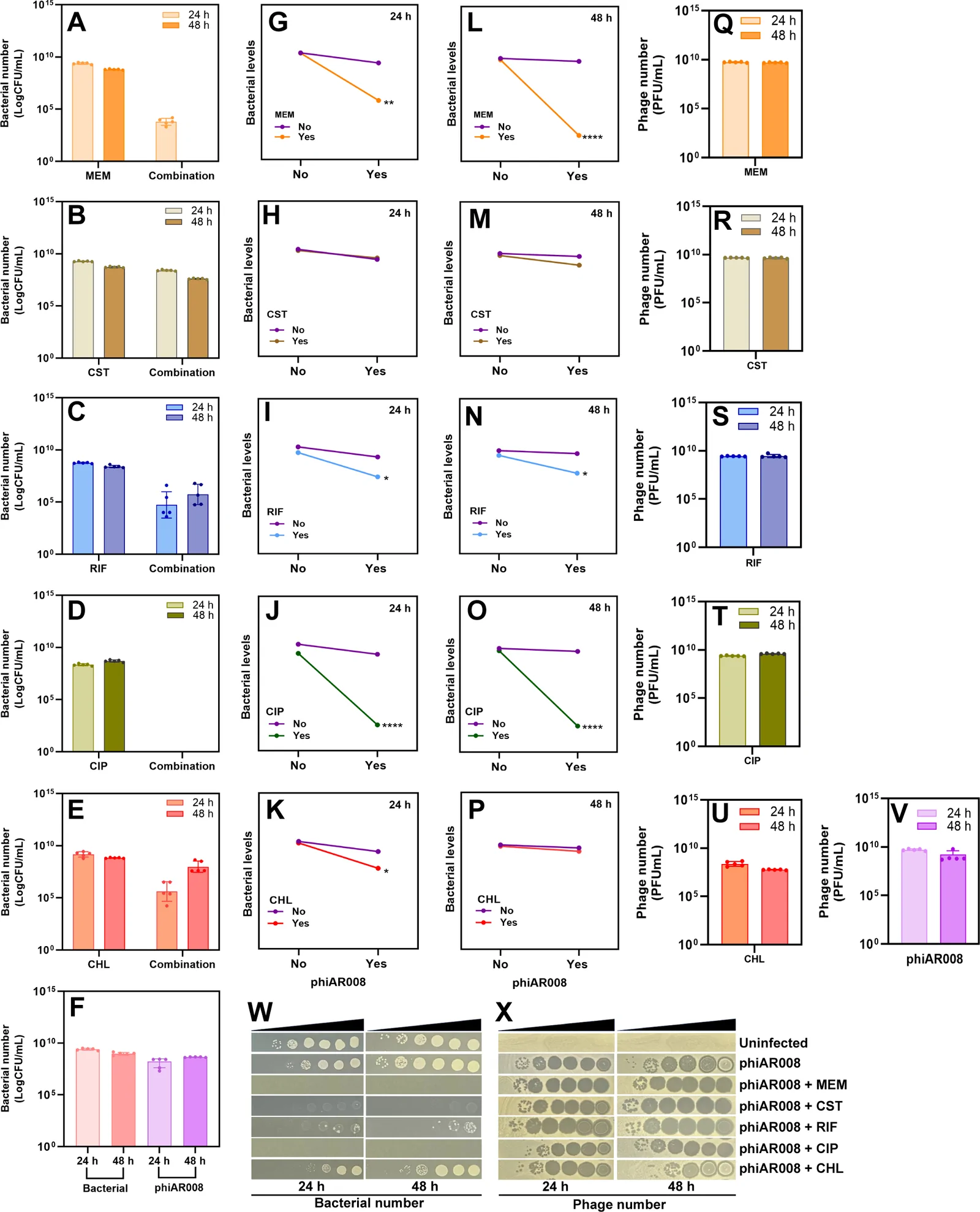
Effects of phage-antibiotic combinations on the CRAB growth and synergistic interactions. Bacterial growth was monitored over 24 and 48 h in the presence or absence of phage and antibiotics (**A–F**). Synergistic interactions were evaluated using interaction plots (**G–P**). Bacterial cells were cultured to the logarithmic phase before treatment with phage (MOI of 0.01) in combination with five antibiotics representing different mechanisms of action: (**A**) the cell wall synthesis inhibitor MEM (1 × MIC), (**B**) the membrane disruptor CST (0.5 × MIC), (**C**) the RNA transcription inhibitor RIF (0.5 × MIC), (**D**) the DNA replication inhibitor CIP (1 × MIC), and (**E**) the protein synthesis inhibitor CHL (0.5 × MIC), compared with phage alone and the untreated control (**F**). Interaction plots showing the combined effects of phage phiAR008 with each antibiotic (MEM, CST, RIF, CIP, and CHL) at 24 h (**G–K**) and 48 h (**L–P**) are presented. Phage titers (log PFU/mL) in combination with each antibiotic at 24 and 48 h (**Q–U**) were compared with phage alone (**V**). Bacterial colony counts were determined by dilution plating on TSA at 24 and 48 h (**W**). Phage titers determined by plaque assay under the same conditions are shown in (**X**), comparing phage alone and phage in combination with each antibiotic. Data are presented as the mean ± standard deviation of at least three independent biological replicates. Statistical analysis for panels **G–P** was performed using one-way ANOVA, followed by Tukey’s HSD test (*P ≤ 0.05, **P ≤ 0.01, ***P ≤ 0.001, and ****P ≤ 0.0001). Abbreviations: MEM, meropenem; CST, colistin; RIF, rifampin; CIP, ciprofloxacin; CHL, chloramphenicol.

Although phage phiAR008 exhibited activity against a relatively broad range of *A. baumannii* clinical isolates (Table 1), supporting its potential therapeutic application, its antibacterial efficacy was limited, and marked bacterial regrowth was observed across multiple MOIs (0.001–10) (Fig. 1D). Given that phage-antibiotic combination therapy has emerged as an important strategy for combating multidrug-resistant bacteria (19, 26), we investigated whether phage-antibiotic combination could provide a biologically rational approach to enhance antibacterial efficacy and suppress the evolution of resistance. Here, we evaluated the effects of phage in combination with five antibiotics representing distinct mechanisms of action, including a cell wall synthesis inhibitor (meropenem; MEM), a cell membrane disruptor (colistin; CST), an RNA transcription inhibitor (rifampin; RIF), a DNA replication inhibitor (ciprofloxacin; CIP), and a protein synthesis inhibitor (chloramphenicol; CHL). We first determined the MICs of each antibiotic against CRAB, which were 8 µg/mL for MEM, 2 µg/mL for CST, 1 µg/mL for RIF, 16 µg/mL for CIP, and 256 µg/mL for CHL. To assess phage-antibiotic synergy, we then combined phage at a low MOI of 0.01, which alone did not effectively kill the CRAB, with either the MIC or 1/2 × MIC of each antibiotic. For this, the number of viable bacteria was quantified at 24 and 48 h in the presence or absence of phage and antibiotic, and the resulting interaction plots (26) were used as a proxy for combinatorial activity (Fig. 2). Our results showed that the combination of phage phiAR008 at an MOI of 0.01 with 1/2 × MIC CST (Fig. 2B) or 1/2 × MIC CHL (Fig. 2E) did not significantly change bacterial counts compared with either phage (Fig. 2F) or antibiotic alone over the 48-h observation period, indicating no synergistic effect (Fig. 2H and M). In contrast, the combination of phiAR008 with MEM (1 × MIC) significantly reduced CRAB within 24 h and completely eliminated the bacteria by 48 h (Fig. 2A and W), showing synogram patterns (Fig. 2G and L). Similarly, the combination with CIP (1 × MIC) resulted in complete bacterial eradication over the same period (Fig. 2D and W), indicating strong synergistic antibacterial activity (Fig. 2J and O). A similar but not identical effect was observed with 1/2 × MIC RIF (Fig. 2C), where the number of viable bacteria decreased significantly by 3 log CFU/mL, indicating slight synergistic antibacterial activity (Fig. 2I and N).

Since phiAR008 infected several clinical CRAB isolates (Table 1), we next investigated whether the observed phage-antibiotic synergy could be generalized to other susceptible strains identified in the host range analysis. Three representative CRAB isolates were selected based on their phage infectivity, as determined by the efficiency of plating (EOP): PT003, which exhibited high phage productivity comparable to the parental host PT061, and PSU104 and PSU131, which exhibited low EOP values (Table 1). The combinations of phiAR008 with either MEM or CIP produced significant synergistic antibacterial activity against PT003 (Fig. S2A, B, E and F). In contrast, no synergistic effect was observed in PSU104 (Fig. S2I through L) or PSU131 (Fig. S2C, D, G and H), both of which exhibited low phage infectivity. These findings suggest that the phage-antibiotic synergistic effect can extend to other susceptible CRAB isolates but is dependent on the ability of phiAR008 to productively infect the target bacterial strain.

We further quantified phage titers to determine whether antibiotics affected phage production. The results showed that phage titers were similar between the phage-antibiotic combinations (Fig. 2Q through U) and phage alone (Fig. 2V), indicating that these antibiotics did not interfere with phage replication under antibiotic exposure. Notably, the combinations of phage with MEM and CIP showed no bacterial regrowth, as evidenced by clear zones without CRAB regrowth (Fig. 2X). In contrast, the combinations of phage phiAR008 with CST, RIF, and CHL appeared unable to suppress the development of phage-resistant populations in the clear zone (Fig. 2X). These findings suggest that phage combinations with MEM and CIP, which exhibited strong synergistic effects, may effectively prevent the emergence of phage resistance; however, further investigation is needed to confirm this potential.

### Antibiotic-induced morphological changes increased plaque sizes in the presence of sublethal antibiotic concentrations

**Fig 3:**
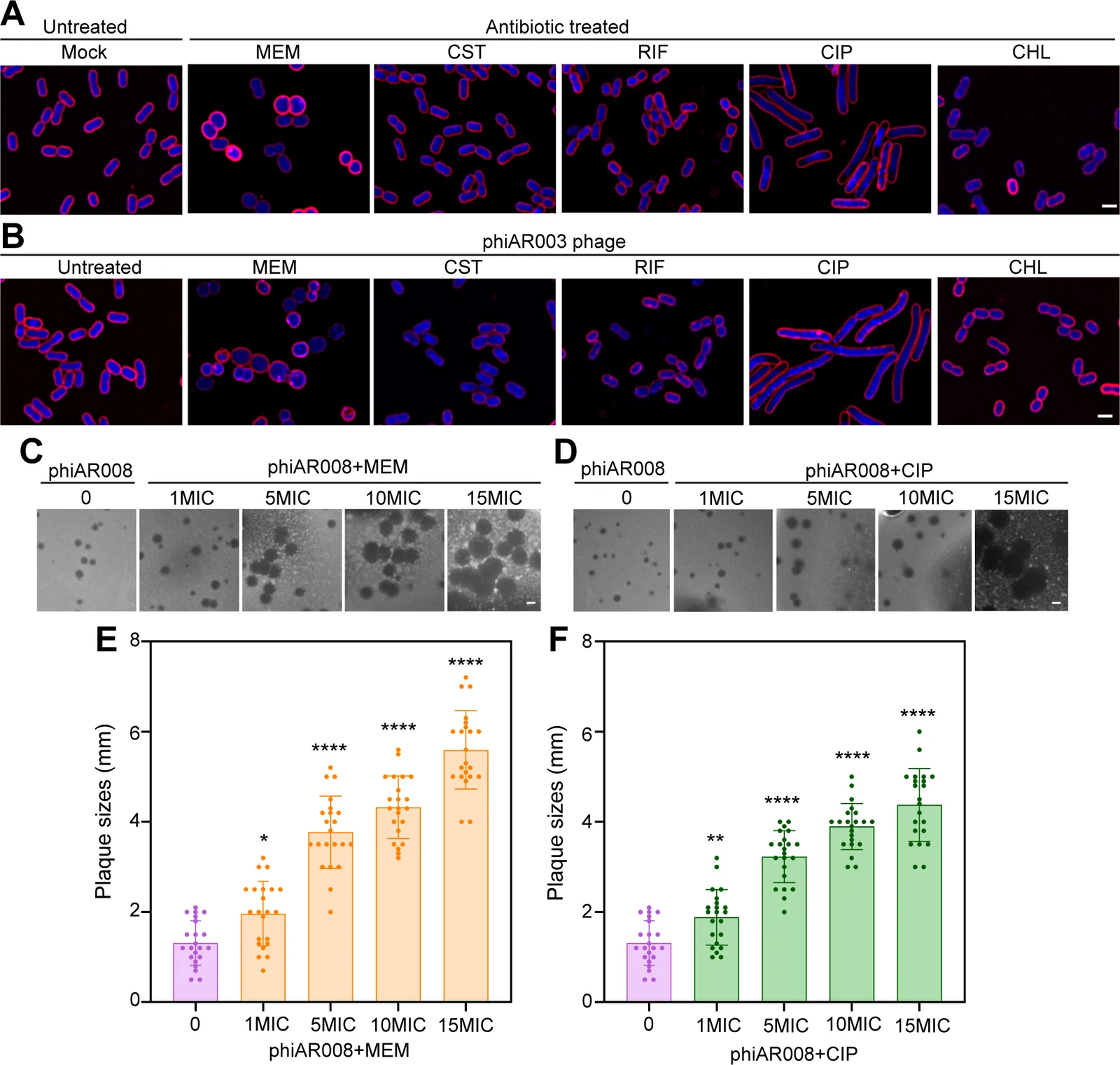
Morphological changes in CRAB following treatment with antibiotics targeting different mechanisms of action and the enhanced plaque formation of phage phiAR008. The BCP was examined using a fluorescence microscopy-based method to assess morphological changes after treatment with MEM, CST, CHL, RIF, and CIP. The method revealed marked morphological alterations in CRAB cells treated with MEM and CIP. CRAB cells were treated with MEM, CST, and CHL at 5 × MIC, RIF at 1 × MIC, and CIP at 2 × MIC, incubated at 37°C for 1 h, and then stained with FM4-64 to visualize the cell membrane (red) and DAPI to stain the nucleoid (blue). Scale bar = 1 µm (**A**). For phage-antibiotic treatment, bacterial cells were grown to the logarithmic phase, treated, incubated at 37°C for 1 h, and infected with phage phiAR008 at an MOI of 0.01 for 20 min (**B**). The treated cells were then stained with FM4-64 (red) and DAPI (blue) and observed by fluorescence microscopy. Plaque size of phiAR008 increased with increasing concentrations of MEM (1, 5, 10, and 15 × MIC), resulting in altered plaque morphology (**C and E**). Similarly, plaque size of phiAR008 increased with increasing concentrations of CIP (1, 5, 10, and 15 × MIC), also resulting in altered plaque morphology (**D and F**). Scale bar = 1 mm. Data are presented as plaque diameter (mm), expressed as the mean ± standard deviation of at least three independent biological replicates. Statistical analysis for panels E and F was performed using one-way ANOVA, followed by Tukey’s HSD test to compare MEM- and CIP-treated groups with the untreated infected control group (*P ≤ 0.05, **P ≤ 0.01, ***P ≤ 0.001, and ****P ≤ 0.0001).

The efficacy of PAS is supported by multiple complementary mechanisms that extend beyond additive killing, and our results also demonstrated strong synergistic activity for the combinations with MEM and CIP (Fig. 2). Previous studies have shown that sublethal antibiotic exposure can induce bacterial elongation in response to PIP and CIP, as well as spheroid cell morphology in response to MER (38), alter cell surface architecture, and increase metabolic activity, thereby enhancing phage adsorption and intracellular replication (16, 24, 25). Here, using bacterial cytological profiling (BCP) (23), we first investigated how bacterial morphology changed following exposure to different antibiotics in the absence or presence of phage. To examine morphological alterations in CRAB cells, we selected five antibiotics with distinct mechanisms of action: MEM as a cell wall synthesis inhibitor, CST as a cell membrane disruptor, RIF as an RNA transcription inhibitor, CIP as a DNA replication inhibitor, and CHL as a protein synthesis inhibitor. As expected, CRAB cells treated with MEM and CIP exhibited marked morphological alterations, with MEM-treated cells showing a rounded phenotype and CIP-treated cells displaying pronounced filamentation as shown in Fig. 3 (38). In contrast, cells treated with CST, RIF, and CHL largely retained their rod-shaped morphology, similar to that of the control group, indicating that these antibiotics did not induce obvious morphological changes (Fig. 3A). To further determine whether phage-antibiotic combinations affected bacterial morphology, CRAB cells were co-treated with phage phiAR008 and antibiotics. As shown in Fig. 3B, no additional morphological changes were observed in the combination treatments compared with antibiotic treatment alone. These results suggest that the presence of phage does not further modify antibiotic-induced morphological alterations.

To systematically evaluate PAS, we tested whether antibiotics from different classes could increase the radii of lysis plaques formed by phage phiAR008 on a CRAB lawn. The antibiotics selected in this study induced either bacterial filamentation through inhibition of cell division (CIP), cell bloating (MEM), or no obvious morphological change (CST, RIF, and CHL). To initially assess the effect of antibiotic-induced changes in cell shape on plaque size, we measured the radii of plaques formed by phage in the presence of 10 × MIC of each antibiotic. Plaques were generated using a standard double-layer agar assay in the presence or absence of the drugs. The results showed that MEM- and CIP-treated CRAB cells significantly increased plaque radii to approximately 3.2–3.5 mm on average, whereas CST, RIF, and CHL had no such effect, as evidenced by plaque sizes comparable to those observed in the untreated control (Fig. S3). We then selected MEM and CIP for further analysis to determine whether the synergistic interaction between phage phiAR008 and antibiotics was dose-dependent. To this end, agar overlay assays were performed across a range of antibiotic concentrations (1 × MIC, 5 × MIC, 10 × MIC, and 15 × MIC), and the radii of the resulting plaques were measured. The results showed that MEM-treated CRAB, which induces spheroid cell morphology (Fig. 3A), significantly increased the plaque size of phage phiAR008 relative to the untreated control, with a clear dose-dependent increase as the antibiotic concentration increased (Fig. 3C and E). The mean plaque diameter in the absence of antibiotics was 1.314 ± 0.495 mm, whereas treatment with MEM increased plaque diameters to 1.964 ± 0.717 mm, 3.768 ± 0.802 mm, 4.323 ± 0.693 mm, and 5.595 ± 0.871 mm at 1 × MIC, 5 × MIC, 10 × MIC, and 15 × MIC, respectively. Similarly, CIP treatment, which induces bacterial filamentation (Fig. 3A), also led to a marked concentration-dependent increase in plaque size (Fig. 3D and F). The corresponding plaque diameters were 1.882 ± 0.615 mm, 3.227 ± 0.576 mm, 3.895 ± 0.512 mm, and 4.373 ± 0.807 mm across the increasing antibiotic concentrations. Here, our findings indicate a dose-dependent synergistic interaction between antibiotics and phage phiAR008. The results suggest that antibiotic-induced morphological changes in bacterial cells play a critical role in modulating plaque size. Specifically, MEM-induced spheroid cell morphology and CIP-induced filamentation both contributed to increased plaque size in a concentration-dependent manner, with MEM being a β-lactam antibiotic and CIP a DNA gyrase inhibitor known to induce filamentation through activation of the SOS response. In contrast, antibiotics such as CST, RIF, and CHL, which do not substantially alter bacterial cell morphology, had no apparent effect on plaque size.

### MEM- and CIP-induced enlargement of treated CRAB cells may enhance phage adsorption and early intracellular phage accumulation

**Fig 4:**
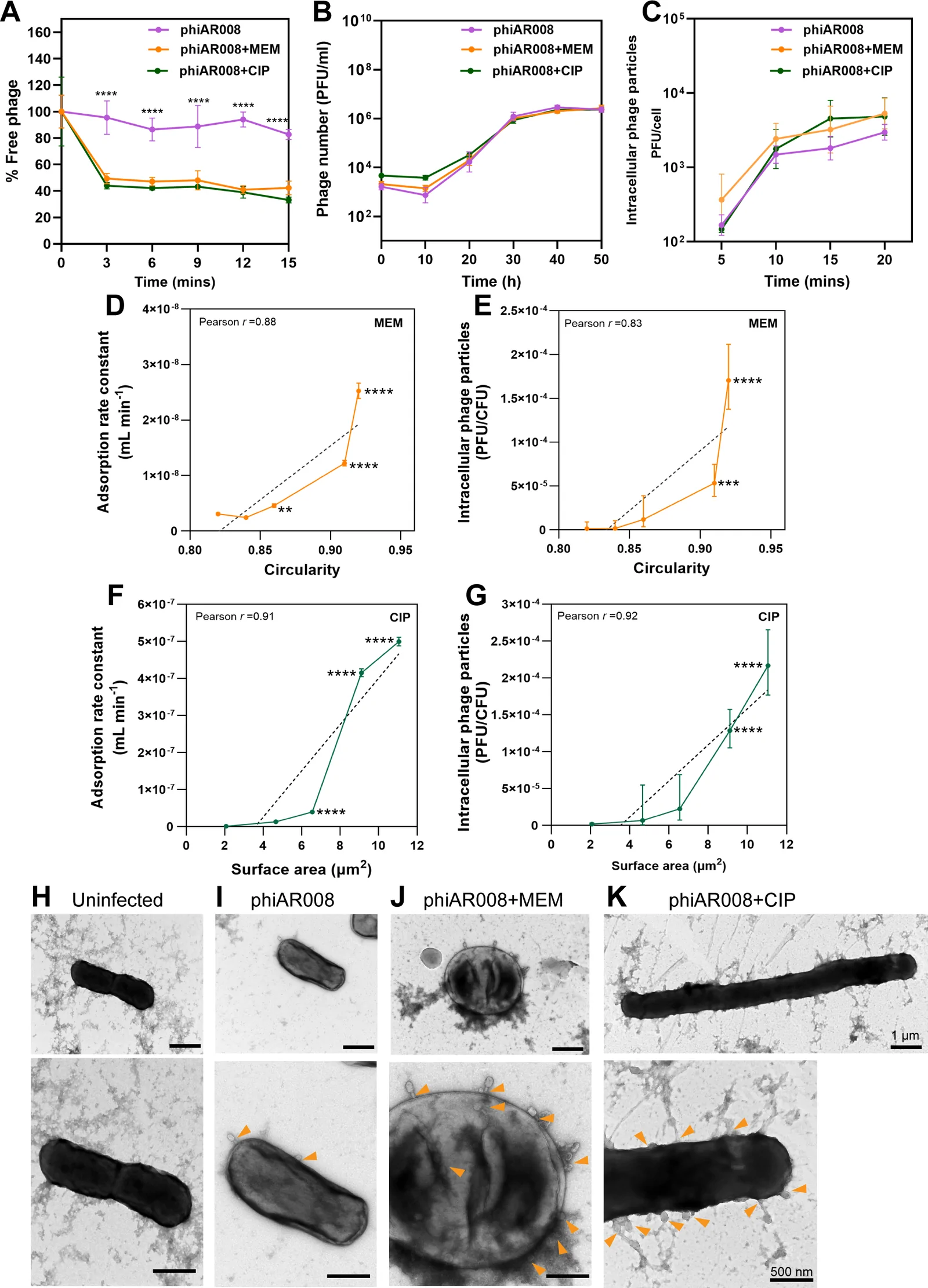
Effects of meropenem (MEM)- and ciprofloxacin (CIP)-induced morphological changes on adsorption and intracellular accumulation of phage phiAR008 in CRAB PT061 cells. CRAB PT061 cells were pretreated with MEM (5 × MIC) or CIP (2 × MIC) for 1 h at 37°C prior to infection with phiAR008 at an MOI of 0.01. The percentage of free phage particles remaining in the supernatant during the adsorption period was determined every 3 min for 15 min (**A**). The one-step growth curve of phiAR008 in the presence of MEM or CIP was performed as part of the adsorption assay, and free phage particles were measured every 10 min up to 50 min (**B**). Intracellular phage particles recovered from infected cells are shown in panel **C**. Correlations between antibiotic-induced morphological alterations and phage infection parameters were further evaluated. Under MEM treatment, increased bacterial circularity was positively associated with the phage adsorption rate constant (**D**) and intracellular phage particles (**E**). Under CIP treatment, enlargement of bacterial surface area was positively associated with the phage adsorption rate constant (**F**) and intracellular phage particles (**G**). Transmission electron microscopy images showing increased virion attachment to untreated CRAB PT061 cells (**I**) compared with pretreated with MEM (**J**) or CIP (**K**). CRAB cells were treated with antibiotics for 30 min at 37°C prior to infection at an MOI of 10 for 15 min. Uninfected cells (**H**), untreated infected cells (**I**), and antibiotic-treated infected cells (**J and K**) were then stained with uranyl acetate and examined by TEM. Orange arrowheads indicate adsorbed phage particles on the bacterial surface. Scale bar: 1 μm and 500 nm. Data are presented as the mean ± standard deviation from at least three independent biological replicates. Statistical analyses for panels **A**, **D**, **E**, **F**, and **G** were performed using one-way ANOVA, followed by Tukey’s HSD test to compare MEM- and CIP-treated groups with the untreated infected cell group (**P ≤ 0.01, ***P ≤ 0.001, and ****P ≤ 0.0001).

Since we previously observed that the combination of phage phiAR008 with MEM- or CIP-treated CRAB completely eradicated the bacterial population and given the strong correlation between phage phiAR008 propagation and the presence of MEM or CIP, it was therefore important to investigate whether the enlargement of MEM- or CIP-treated cells contributed to bacterial eradication through enhanced phage adsorption or intracellular replication. To investigate the kinetics of phage adsorption to the bacterial cells, CRAB cells were pretreated with MEM or CIP for 1 h prior to infection at an MOI of 0.01. The results showed that phage adsorption occurred significantly faster in MEM- or CIP-treated CRAB cells than in the untreated control. Approximately 50% of phages were adsorbed within 3 min, and this level was maintained for up to 15 min, whereas adsorption in the untreated condition remained low at around 10% throughout the same period (Fig. 4A). Consistently, the adsorption rate constants (*k*) were significantly higher in the MEM- and CIP-treated groups, at 7.07 × 10^−9^ mL/min and 8.23 × 10^−9^ mL/min, respectively, compared with 5.30 × 10^−10^ mL/min in the untreated control. We further examined whether cell enlargement induced by MEM and CIP could enhance phage adsorption on the CRAB surface using transmission electron microscopy (TEM) (39). The CRAB cells were treated with each antibiotic for 30 min prior to infection with phage phiAR008 at an MOI of 10. TEM revealed that MEM-treated cells exhibited a spheroid morphology (Fig. 4J), whereas CIP-treated cells displayed pronounced elongation (Fig. 4K), in contrast to untreated infected cells (Fig. 4I) and untreated uninfected cells (Fig. 4H), which retained a rod-shaped morphology. These observations were consistent with the bacterial cytological profiling results obtained by fluorescence microscopy (Fig. 3A and B). As shown in Fig. 4J and K, enlarged cells treated with MEM or CIP were densely covered with attached phiAR008 particles. In CIP-treated cells, phage particles were frequently observed attached via the head region, whereas in MEM-treated cells, the majority of phage particles were attached to the bacterial surface via the baseplate of the tail. In contrast, only a few phage particles were observed on the surface of untreated infected cells (Fig. 4I).

An additional question was whether antibiotic-induced morphological changes affect the latent period, defined as the interval between infection and release of progeny phage, as well as the burst size, defined as the number of phages produced per infected cell. To address this, we measured the burst size of phage phiAR008 in untreated cells and compared it with that in MEM- or CIP-treated conditions using a classical one-step growth curve assay. All experiments were performed using CRAB cells pretreated with MEM or CIP for 1 h prior to infection at an MOI of 0.01. Unexpectedly, neither the latent period nor the burst size differed significantly among the untreated, MEM-treated, and CIP-treated groups. However, phage titers at 10 min post-infection were modestly higher in antibiotic-treated cells than in untreated cells (Fig. 4B), suggesting that antibiotic-induced cell enlargement may facilitate the early stages of phage infection without altering overall phage replication kinetics. We therefore quantified intracellular phage production throughout the infection cycle. Intracellular phage particles increased dramatically between 5 and 10 min post-infection in both MEM- and CIP-treated cells and remained relatively stable thereafter. In contrast, untreated cells consistently produced lower intracellular phage titers than antibiotic-treated cells throughout the experiment (Fig. 4C). These findings suggest that antibiotic-induced morphological changes may enhance the early accumulation of intracellular phage particles without affecting the latent period or burst size.

We next investigated whether the extent of antibiotic-induced cell enlargement influences phage adsorption efficiency and the initiation of intracellular phage infection. CRAB cells were pretreated for 1 h with MEM or CIP at 1×, 2×, 5×, or 10 × MIC, followed by infection with phiAR008 at an MOI of 0.0001 for 6 min. These conditions were selected to examine the early stages of phage infection while minimizing secondary rounds of infection. MEM treatment induced progressive cell enlargement, whereas CIP treatment resulted in pronounced filamentation, leading to dose-dependent increases in bacterial surface area. Correspondingly, the phage adsorption rate constant (*k*) increased significantly with increasing bacterial surface area in both MEM-treated (Fig. 4D) and CIP-treated cells (Fig. 4F) relative to untreated cells. Pearson correlation analysis demonstrated a strong positive relationship between bacterial surface area and the phage adsorption rate constant in both MEM-treated (*r* = 0.88) and CIP-treated (*r* = 0.91) cells. We next quantified intracellular phage accumulation during the early stage of infection. In MEM-treated cells, intracellular phage levels increased proportionally with bacterial surface area (Fig. 4E). Likewise, CIP-induced filamentation resulted in a surface area-dependent increase in intracellular phage accumulation (Fig. 4G). Consistent with these observations, intracellular phage production was positively correlated with bacterial surface area in both MEM-treated (*r* = 0.83) and CIP-treated (*r* = 0.92) cells. Hence, these findings demonstrate that increases in bacterial surface area enhance both phage adsorption efficiency and the early accumulation of intracellular phage particles, indicating that antibiotic-induced morphological changes facilitate the initial stages of productive phage infection.

Overall, our results indicate that antibiotic-induced bacterial morphology does not significantly alter the latent period or burst size of phiAR008 (Fig. 3 and 4). Instead, the enhanced antibacterial activity of the phage-antibiotic combinations appears to result primarily from increased phage adsorption and more efficient intracellular phage accumulation during the early stages of infection rather than increased phage production per lytic cycle. These effects likely contribute to the more rapid and efficient eradication of CRAB cells observed during combination treatment.

### The combinations of phage phiAR008 with MEM or CIP were superior to single-agent treatments against the CRAB both *in vitro* and *in vivo*

**Fig 5:**
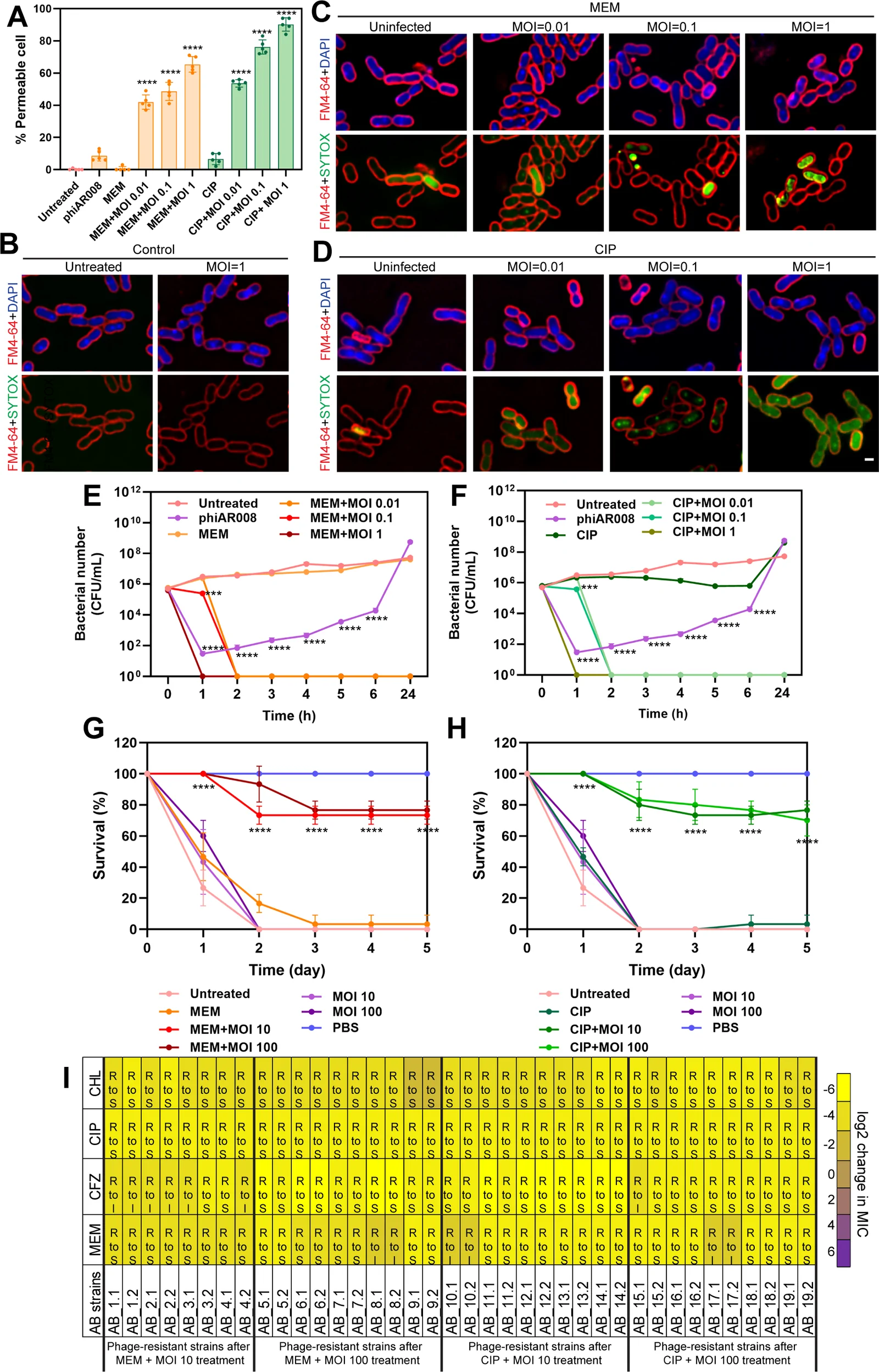
Synergistic antibacterial activity of phage phiAR008 at different MOIs in combination with MEM and CIP against CRAB *in vitro* and *in vivo*. Percentage of bacterial cells exhibiting increased membrane permeability under the indicated conditions, including the uninfected control, antibiotic treatment alone (MEM and CIP), and combination treatment with phage phiAR008 at MOIs of 0.01, 0.1, and 1 (**A**). Bacterial cultures were grown to the log phase prior to treatment and incubated at 37°C for 20 min under the specified conditions. Representative fluorescence microscopy images of bacterial cells under untreated conditions and after phage infection at an MOI of 1 (**B**). Membrane permeability of CRAB cells after treatment with MEM (**C**) or CIP (**D**) in combination with phage phiAR008 at different MOIs, detected by SYTOX Green staining. Time-kill kinetics were performed to determine whether the increased membrane permeability observed in the combination treatments with MEM (**E**) and CIP (**F**) translated into enhanced bacterial killing, measured by bacterial viability (CFU/mL) at 0, 1, 2, 3, 4, 5, 6, and 24 h, compared with single-agent treatments and untreated bacteria. Survival of infected *G. mellonella* larvae for 5 days following treatment with antibiotics alone, phage phiAR008 alone (MOIs of 10 and 100), or combined phage-antibiotic therapy using MEM (**G**) or CIP (**H**) at MOIs of 10 and 100. PBS-treated groups served as negative controls. Consistent with the *in vitro* antibacterial activity, the combination treatments resulted in improved survival compared with individual treatments. Heatmap illustrating changes in minimum inhibitory concentration (MIC) values of antibiotics (MEM, CFZ, CIP, and CHL) across different phage-resistant isolates. Colors indicate log2 fold changes in MIC values, and susceptibility status is denoted as susceptible (S), intermediate (**I**), or resistant (R) (**I**). Statistical analysis for panels A, E, F, G, and H was performed using one-way ANOVA, followed by Tukey’s HSD test to compare the untreated group with the treatment groups (****P ≤ 0.0001). All experiments were conducted using at least three biological replicates. MEM, meropenem; CFZ, ceftazidime; CIP, ciprofloxacin; CHL, chloramphenicol.

To explore the bactericidal activity of phage phiAR008-antibiotic combinations, BCP was assessed using a fluorescence microscopy-based method (22) to evaluate bacterial viability and membrane disruption in CRAB following exposure to the combinations and single-agent treatments. In order to investigate this phenomenon and determine whether the synergistic behavior between antibiotics and phage was dose-dependent, CRAB cells were incubated with the indicated treatments for 20 min in the presence of fluorescent dyes and then examined by fluorescence microscopy. Fluorescence microscopy showed that FM4-64, DAPI, and SYTOX Green were used to visualize the cell membrane boundary, nucleic acids, and membrane integrity, respectively. DAPI stained all cells with intact membranes, whereas SYTOX Green selectively labeled cells with compromised membrane integrity (40). Following treatment of CRAB cells with phage phiAR008 in combination with MEM or CIP, SYTOX Green-positive cells increased to 41.9%, 48.7%, and 65.5% at MOIs of 0.01, 0.1, and 1, respectively, for the phage-MEM combination (Fig. 5A and C). In contrast, only 8.5%, 0.9%, and 0.3% of cells were stained in the phage-only, MEM-only, and untreated control groups, respectively (Fig. 5B), indicating significantly fewer membrane-compromised cells than in the combination treatment. Similarly, the phage-CIP combination induced a marked, MOI-dependent increase in SYTOX Green fluorescence, with 53.6%, 76.4%, and 90.2% of cells stained at MOIs of 0.01, 0.1, and 1, respectively (Fig. 5A and D), compared with the untreated control and single-agent treatments (Fig. 5B and D). These findings indicate increased membrane permeability and greater membrane disruption than that observed under the other conditions.

To further confirm the synergistic activity of phiAR008 with MEM or CIP, time-kill assays were performed *in vitro* against CRAB treated with antibiotics combined with different phage doses. As shown in Fig. 5E and F, the combination of MEM or CIP with phage at an MOI of 1 completely eliminated CRAB within 1 h, whereas single phage treatment at the same MOI caused an initial significant reduction in viable bacteria during the first hour, followed by marked regrowth up to 24 h, similar to the untreated control and single-antibiotic groups. In addition, phage treatment at an MOI of 0.01 in combination with MEM or CIP did not significantly reduce viable CRAB within the first hour of treatment, whereas increasing the MOI to 0.1 resulted in a significant reduction in viable bacteria after 1 h. However, both combinations exhibited progressively stronger synergistic bactericidal activity after 2 h and ultimately achieved complete bacterial eradication over time (Fig. 5E and F). These findings indicate that the antibiotic-phage combinations effectively and synergistically eliminate CRAB, likely through enhanced membrane disruption (Fig. 5A through D), leading to bacterial cell death. Collectively, these results demonstrate strong synergistic bactericidal activity of phiAR008 with MEM or CIP, with higher phage concentrations associated with greater membrane permeability and disruption in CRAB, thereby providing insight into the possible mechanism of action of PAS.

We then evaluated the therapeutic efficacy of the phiAR008-MEM or phiAR008-CIP combination *in vivo* using a *G. mellonella* infection model. Larvae were infected with the CRAB for 1 h prior to treatment with phiAR008 in combination with MEM or CIP, and the results were compared with those of single-agent treatments and the PBS control. The survival curves showed that the infected group caused approximately 70% mortality within 1 day and complete mortality after 2 days (Fig. 5G and H). In the single-agent treatment groups, survival decreased to approximately 45–60% by day 1 and reached 0% by day 2. In contrast, treatment with phiAR008 combined with MEM or CIP significantly reduced larval mortality compared with monotherapy at MOIs of 10 and 100 (Fig. 5G and H). Most combination groups maintained survival rates of around 80%, while the phiAR008-MEM treatment at an MOI of 100 yielded approximately 90% survival on day 1. Furthermore, survival remained stable after 2 days, with approximately 75–80% of larvae surviving throughout the observation period. These results indicate that phiAR008-MEM or phiAR008-CIP combination therapy was significantly more effective than phage monotherapy in treating the CRAB infection in *G. mellonella*. Previous studies of phage-antibiotic combinations in mice (15, 19) have similarly demonstrated enhanced antibacterial efficacy and increased antibiotic susceptibility. Here, we further randomly screened phage-resistant mutants from larvae treated with all combinations across the experiments. Interestingly, the phage-resistant isolates exhibited restored susceptibility to multiple antibiotics, including MEM, CFZ, CIP, and CHL, compared with the wild-type strains (Fig. 5I). Among phage-resistant isolates derived from the phiAR008–MEM combination, 66.7% (12/18) showed a 5–6-log reduction in CFZ MIC, becoming susceptible, while 33.3% (6/18) showed a 4-log reduction, shifting to the intermediate category. In addition, 88.9% (4/11) of isolates showed a 4–5-log reduction in MEM resistance, and all phage-resistant isolates showed a 5-log reduction in CIP resistance and a 4–5-log reduction in CHL resistance (Fig. 5I). Similar trends were observed in phage-resistant isolates obtained from the phiAR008-CIP combination, which exhibited reduced resistance to MEM, CFZ, CIP, and CHL by 3–6-log reductions (Fig. 5I). Overall, these findings suggest that PAS not only enhances bacterial eradication but also limits the emergence of antibiotic and phage resistance, offering a promising strategy for managing difficult-to-treat infections.

## DISCUSSION

Phage-antibiotic combination therapy has emerged as a critical strategy against multidrug-resistant *A. baumannii*, offering a synergistic approach to enhance antibacterial efficacy and suppress resistance evolution (15). By engaging in phage-antibiotic synergy (PAS), these agents act cooperatively to lower required antibiotic concentration, exploit evolutionary trade-offs, and resensitize bacteria to traditional antibiotics (41). This dynamic is particularly advantageous for dismantling recalcitrant biofilm-associated infections where monotherapies frequently fail, as demonstrated across diverse *in vitro*, *ex vivo*, and *in vivo* models (16, 17, 42). However, while the complementary mechanisms of PAS hold significant clinical promise, translating these effects into practice requires the careful selection of partner antibiotics, as synergistic outcomes are highly dependent on the specific antibacterial mechanisms of action involved (26).

In this study, we isolated the phage phiAR008, classified within the genus *Hadassahvirus* (Fig. 1E and F), which demonstrated infectivity against multiple clinical CRAB strains (Table 1). Despite its ability to infect multiple clinical CRAB isolates, its bactericidal efficacy as a monotherapy was limited. Treatment across various MOIs (0.001–10) produced a modest initial reduction in viable bacterial counts at 24 h, followed by substantial regrowth by 48 h (Fig. 1D). This rapid emergence of host resistance is consistent with observations of other *Hadaddahvirus* members, including phages Maestro, AC4, and Navy71 (20, 37). Ultimately, this resurgence underscores the therapeutic limitations of standalone phage treatment and highlights the necessity for combinatorial approaches. To address this, we evaluated phiAR008 at a low infectious dose (MOI = 0.01) alongside sublethal concentrations of five antibiotics representing distinct mechanisms of action: MEM; cell wall, CST; cell membrane, RIF; RNA transcription, CIP; DNA replication, and CHL; protein synthesis. Interaction profiling (synograms) revealed significant synergy when phiAR008 was combined with either MEM or CIP, both of which achieved complete eradication of CRAB by 48 h. Conversely, CST and CHL were non-synergistic, and RIF showed only marginal synergy (Fig. 2). These findings align strongly with recent literature demonstrating potent synergy between specific phages and beta-lactam antibiotics. For instance, pairing phages (such as pB3074 or pB23) with cefotaxime or meropenem yields profound synergistic effects against CRAB, not only clearing planktonic cells but also eradicating mature biofilms and successfully treating wound infections in *ex vivo* porcine models (16, 17). Similarly, the synergy we observed with CIP parallels recent *in vivo* reports in which phage-CIP co-administration yielded outcomes significantly superior to either monotherapy, explicitly enhancing survival rates, restoring T-cell homeostasis, and markedly attenuating pro-inflammatory cytokine levels (43, 44). Notably, because CIP inhibits bacterial DNA replication, it was anticipated to interfere with phage proliferation. However, genomic analysis revealed that phiAR008 encodes key independent DNA replication elements such as a DNA polymerase sliding clamp (*gp7*), an RNA polymerase-binding protein (*gp8*), deoxynucleotide monophosphate kinase (*gp66*), and dCMP deaminase (*gp125*) (Table S1). These elements may enable phiAR008 to maintain robust amplification even in CIP-treated cells, achieving replication levels comparable to untreated controls (Fig. 2T and V). Taken together, our findings indicate that phiAR008 combined with either MEM or CIP represents a highly promising, mechanistically driven strategy for the management of CRAB infections.

Mechanistically, the success of PAS is supported by complementary processes that extend beyond simple additive killing. Sublethal antibiotic exposure, particularly with β-lactams (MEM), which alter cell surface architecture, or fluoroquinolones (CIP), which induce the SOS response, can trigger distinct physiological shifts in bacteria, thereby enhancing phage adsorption and intracellular replication (16, 38). Consistent with this, treating CRAB with MEM or CIP induced distinct spheroid and filamentous morphologies, whereas treatment with CST, RIF, or CHL yielded cell shapes comparable to the untreated control (Fig. 3A). Notably, phage phiAR008 alone did not elicit these morphological changes (Fig. 3B). To determine whether this synergy operates through universal or class-specific mechanisms, we evaluated the effect of sublethal antibiotic concentrations on phiAR008 plaque sizes. Exposure to MEM and CIP induced a dose-dependent enlargement of plaques, indicative of robust PAS (Fig. 3C through F), whereas CST, RIF, and CHL exerted no effect on plaque size (Fig. S2). These findings, mirroring prior validation with diverse phages such as T5 and T7, further establish plaque size modulation as a reliable, standardized metric for quantifying PAS interactions (24, 25). Given that synergistic effects were exclusively observed alongside antibiotic-induced morphological changes (Fig. 2 and 3), we hypothesize that PAS is primarily driven by these structural alterations, which facilitate initial phage adsorption rather than by the specific molecular targets of the antibiotics. To test this hypothesis, we quantified phage adsorption rates (Fig. 4A), one-step growth kinetics (Fig. 4B), and intracellular viral particle production (Fig. 4C) in CRAB cells that were pretreated with MEM or CIP to induce cellular enlargement, comparing these results to untreated controls.

Our findings demonstrate that an increase in bacterial cell surface area is associated with enhanced phage adsorption. Specifically, the adsorption rate constants for meropenem (MEM)- and ciprofloxacin (CIP)-treated cells were significantly higher than those of untreated cells, indicating more rapid attachment of phiAR008 to antibiotic-treated bacteria (Fig. 4A, D and F). Transmission electron microscopy further supported these findings, revealing that the enlarged antibiotic-treated cells were densely decorated with adsorbed phiAR008 particles (Fig. 4H through K). In contrast, the antibiotic-induced filamentous and spheroid morphologies did not substantially alter phage burst size or the number of intracellular phage particles produced at the completion of the replication cycle (Fig. 4B and C). Because neither the productivity nor the completion of the intracellular replication cycle differed appreciably among the distinct cell morphologies, we hypothesized that the observed synergy was primarily attributable to changes during the early stages of infection rather than increased phage production. To further investigate this hypothesis, we examined whether bacterial cell surface area influences phage adsorption and early-stage infection. We found that both the adsorption rate and the accumulation of intracellular phage particles during the early phase of infection increased with increasing cell surface area (Fig. 4D–G), whereas the total phage yield at the end of the replication cycle remained largely unchanged. These findings suggest that enlargement of bacterial cells enhances the efficiency of the initial stages of phage infection without substantially affecting the final phage production per infected cell. Interestingly, previous studies have shown that bacterial filamentation can prolong the latent period and increase burst size, a phenomenon explained by the “delayed lysis” hypothesis, in which membrane expansion increases the holin concentration threshold required to trigger host cell lysis (24, 25, 27). In contrast, our data support a distinct mechanism. Enlargement of the bacterial cell appears to enhance the probability of phage-host encounters by increasing the available cell surface area, thereby accelerating phage adsorption and early-stage infection (Fig. 4). This enhancement likely contributes to the more rapid bacterial killing observed during combination treatment and is consistent with the complete bacterial clearance and absence of regrowth in the MEM- and CIP-combination groups (Fig. 2X). Collectively, these findings suggest that antibiotic-induced changes in bacterial morphology can increase the efficiency of early phage-host interactions, thereby enhancing the antibacterial activity of phage-antibiotic combination therapy without altering the overall productivity of individual phage replication cycles.

Our study further demonstrated that the combination of phage phiAR008 and antibiotics exhibited significant synergistic efficacy compared to monotherapy against CRAB both *in vitro* and *in vivo*. The BCP assay successfully elucidated membrane disruption, which likely leads to cellular toxicity and eventual cell death, as membrane permeabilization is a well-established mechanism of antibacterial activity (45, 46). Consequently, the cellular uptake of SYTOX Green indicated that the phage phiAR008-MEM and phiAR008-CIP combinations possess significantly greater membrane-permeabilizing activity than single-agent treatments (Fig. 5A through D), although we were unable to pinpoint the exact macromolecular target. These findings are consistent with the bactericidal activity observed in our time-kill assays, which revealed that the combination treatments completely eradicated the bacteria within 1 h. No viable bacteria were detected for the remainder of the 24-h period, demonstrating that the efficacy of the combinatorial treatment was independent of the initial phage dosage (Fig. 5E and F). In contrast, during phage phiAR008 monotherapy, viable bacterial counts significantly declined at 1 h but were followed by dramatic macroscopic regrowth over the remaining incubation period. Furthermore, bacterial viability during single-agent antibiotic treatment remained comparable to that of the untreated controls (Fig. 5E and F). Together, these findings suggest that MEM- or CIP-induced cell enlargement facilitates faster phage adsorption across the expanded cell surface, precipitating rapid cell death as evidenced by profound membrane permeabilization and the complete loss of bacterial viability.

To determine whether the *in vitro* efficacy of phage-antibiotic combinations translates *in vivo*, we evaluated these strategies using an animal infection model. Our results using the *G. mellonella* model demonstrate that this combination therapy exhibits robust *in vivo* antibacterial activity, highlighting its potential as a viable alternative therapeutic strategy (47, 48). Because the efficacy of the combinatorial treatment was previously shown to be dependent on the initial phage dosage (43), we evaluated the phage phiAR008 combination at higher MOIs of 10 and 100. Our findings reveal that combining phage phiAR008 with either MEM or CIP provides superior protection against CRAB infection compared to monotherapies (Fig. 5G and H), which is consistent with previous *in vivo* preclinical studies (15, 17, 49). The approximately 80% survival rate observed in the combination treatment groups can be attributed to several underlying mechanisms. First, the synergistic treatment effectively reduces the initial bacterial burden; antibiotic-induced cellular enlargement enhances initial phage adsorption and subsequent viral replication, driving efficient bacterial lysis, complete clearance, and reduced infection recurrence. Second, as reported in previous studies, phage infection may alter the bacterial outer membrane, thereby enhancing the pathogen’s susceptibility to antibiotics (18, 26, 50, 51) or causing it to exhibit attenuated virulence (19, 51, 52). Importantly, evidence for *in vivo* PAS continues to grow. Combining phages with antibiotics has been shown to significantly lower bacterial burdens in infection models while promoting the emergence of phage-resistant yet antibiotic-sensitive bacterial populations—an evolutionary trade-off with significant therapeutic potential (15, 19, 20). As evidenced in our study, phage-resistant strains isolated from the larvae following the phiAR008-MEM or CIP combination treatments exhibited restored sensitivity to meropenem, ceftazidime, ciprofloxacin, and chloramphenicol (Fig. 5I). This restoration of antibiotic susceptibility represents another mechanism contributing to the synergistic efficacy of the combinations. However, the exact molecular mechanisms driving this resensitization require further elucidation. Although increased antibiotic sensitivity is frequently associated with phage resistance in *A. baumannii* (19, 51), some studies have reported increased cross-resistance in phage-resistant mutants (53, 54). Thus, antibiotic resensitization is not a guaranteed outcome of phage resistance, as it relies heavily on the specific host, drug, phage, and underlying mutation. While continually applying selection pressure via phage-antibiotic combinations may yield sustained therapeutic benefits even after phage resistance emerges, further studies are required to elucidate these evolutionary mechanisms.

Consequently, phage-antibiotic combination therapy represents a promising therapeutic approach for the treatment of multidrug-resistant infections that are refractory to conventional antibiotic therapy. However, the selection of antibiotic partners should be carefully optimized based on both *in vitro* susceptibility profiles and *in vivo* preclinical contexts. In this study, we characterized phiAR008, a lytic phage that infects diverse CRAB isolates but exhibits relatively low standalone bactericidal efficacy against CRAB strains. We further demonstrated that combining phiAR008 with selected antibiotics resulted in marked synergistic antibacterial activity against CRAB in both *in vitro* and *in vivo* models, achieving substantially greater efficacy than phage treatment alone. Our findings suggest that antibiotic-induced bacterial cell enlargement may enhance initial phage adsorption and facilitate early-stage phage replication, thereby improving bacterial clearance and reducing infection recurrence. However, the precise mechanisms underlying this interaction remain to be elucidated. In addition, phage-resistant isolates recovered following *in vivo* treatment exhibited reduced virulence and altered antibiotic susceptibility. Although these observations are consistent with phenotypic changes associated with phage resistance, the genetic and mechanistic basis of these alterations was not investigated in the present study and warrants further investigation. Collectively, these findings highlight the therapeutic potential of rationally optimized phage-antibiotic combinations as an alternative strategy for the treatment of refractory CRAB infections.

## MATERIALS AND METHODS

### Bacterial strains and growth conditions

In this study, a total of 24 bacterial strains were utilized. These included 18 strains of carbapenem-resistant *A. baumannii* (CRAB), comprising nine clinical isolates from Phatthalung Hospital, nine clinical isolates from Songklanagarind Hospital, and one isolate provided by the Department of Medical Sciences, Ministry of Public Health, Thailand. Reference strains included *A. baumannii* ATCC 19606, *Staphylococcus aureus* ATCC 29312, *Pseudomonas aeruginosa* ATCC 27853, *Klebsiella pneumoniae* ATCC 700603, and *Escherichia coli* ATCC 25922. CRAB strain PT061 served as the primary parental host for phage isolation. To prepare the bacterial inoculum, a single colony cultivated on Trypticase Soy Agar (TSA) was inoculated into 5 mL of Trypticase Soy Broth (TSB) and incubated overnight at 37°C with continuous agitation at 150 rpm. Subsequently, the overnight culture was diluted 1:100 in fresh TSB and incubated under identical conditions (37°C, 150 rpm) for 1 h to achieve the logarithmic growth phase.

### Phage biological characterization

#### Phage isolation, purification, plaque morphology, and propagation

To isolate phages specific to CRAB strain PT061, we employed an enrichment method using influent wastewater sourced from student dormitories at Prince of Songkla University (Hat Yai Campus, Songkhla, Thailand). The wastewater samples were first filtered through a 0.45 µm membrane and mixed directly with TSB powder. A logarithmic-phase culture of the PT061 host strain was added to this mixture, followed by a 24-h incubation at 37°C with shaking at 150 rpm. Post-incubation, the culture was centrifuged, and the clear supernatant was filtered. Phage presence was detected via the double-layer agar method (DLA): the filtrate and host bacteria were combined in 0.35% TSA soft agar, poured over solid TSA plates, and incubated at 37°C for 24 h. Single plaques were selected and purified through at least three consecutive rounds of plating. The final purified phage lysates were resuspended in SM buffer (200 mM NaCl, 10 mM MgSO₄·7H₂O, 50 mM Tris-HCl, pH 7.5) and stored at 4°C. Plaque morphology, encompassing size and phenotypic characteristics, was subsequently documented.

For phage propagation, 200 µL of the host bacterial culture was combined with 50 µL of phage suspension and 5 mL of TSA soft agar. This mixture was then overlaid onto a TSA plate and incubated at 37°C for 24 h. Following incubation, 5 mL of SM buffer was added to the plate, which was then left at room temperature for 5 to 6 h. The bacterial cells were subsequently removed by centrifugation, and the resulting supernatant was filtered through a 0.45 µm membrane filter before being stored at 4°C for subsequent experiments. Phage titration was performed by mixing tenfold serial dilutions of phage stocks with the host bacterial culture, followed by plating onto TSA soft agar. The plates were incubated overnight at 37°C, after which phage quantities were determined by counting plaques and expressed as plaque-forming units per milliliter (PFU/mL).

#### Phage morphology by transmission electron microscopy

Phage suspension was dropped onto the lawn of the parental host and incubated at 37°C for 24 h. The resulting clear was collected, immersed in an SM buffer, and stored at 4°C overnight. Following centrifugation at 5,000 rpm for 5 min, the supernatant was filtered through a 0.45 µm membrane. The concentrated phage suspension was then applied to a copper grid and stained with 2% uranyl acetate solution for 1 min. Then rinsing and blotting, the grids were dried for 10 min. Phage morphology was performed using a Thermo Scientific Talos F200i transmission electron microscope operating at 200 kV.

#### Adsorption

The phage adsorption curve was established to quantify the time required for phage attachment to host cells. Host cells were cultured to the 0.5 McFarland standard. The cultures were then infected with phages at a multiplicity of infection (MOI) of 0.01 and incubated at 37°C. Aliquots of 300 µL were collected at 0, 3, 6, 9, 12, and 15 min and immediately filtered through a membrane with a pore size of 0.45 µm. The concentration of unabsorbed phages in the filtrates was subsequently determined. All experiments were conducted independently in triplicate.

#### One-step growth curve

A one-step phage growth curve was conducted to assess the latent period and burst size of the phage. Five milliliters of host culture at log phase was infected with phage at a multiplicity of infection (MOI) of 0.01 and incubated at 37°C for 10 min to allow phage adsorption. Following centrifugation at 4,500 rpm for 5 min, the supernatant containing free phages was collected to quantify the number of unabsorbed phages. This value was subtracted from the initial phage count to estimate the number of infected cells. The pellet, containing the infected cells, was resuspended in 6 mL of TSB and incubated at 37°C. Samples were taken at 10-min intervals over a period of 120 min and filtered through a 0.45 µm membrane. The phage titer was determined using a spot titer assay via the double-layer agar method. The latent period was defined as the duration from the initiation of the experiment to the onset of phage progeny production. The burst size was calculated by subtracting the number of unabsorbed phages from the average maximum phage yield and dividing the result by the number of infected cells (PFU/CFU). All experiments were performed independently in triplicate.

#### Time-kill assay

To evaluate the bacterial activity of the phage, log-phase CRAB strains PT061 cells were treated with phages at MOIs of 0.001, 0.01, 0.1, 1, and 10. Briefly, bacterial suspensions were cells dispensed into a 96-well microplate and mixed with phages at the indicated MOIs in 200 µL of TSB. Viable bacterial counts were determined at 24 and 48 h post-treatment using the plate counting method. The results were presented as bar graphs to compare bacterial survival among treatment groups. All experiments were performed independently in triplicate.

#### Host range and efficiency of plating (EOP)

A total of 24 bacterial strains were used to evaluate the host range, including 18 CRAB isolates, including nine from Phatthalung Hospital and nine from Songklanagarind Hospital, as well as *A. baumannii* DMST 2071, *A. baumannii* ATCC 19606, *Klebsiella pneumoniae* ATCC 700603, *Staphylococcus aureus* ATCC 29312, *Pseudomonas aeruginosa* ATCC 27853, and *Escherichia coli* ATCC 25922. A modified host range assay was performed using a 96-well microplate. Briefly, 10 µL of bacterial culture was mixed with 50 µL of TSB and 70 µL of 1% low-melt TSA and then allowed to solidify for 15 min. Subsequently, 50 µL of either phage solution or SM buffer (control) was added to each well, followed by incubation at 37°C for 24 h to observe the clear solution. Bacterial strains that were susceptible in the host range assay were further evaluated for EOP using the double-layer agar plaque assay. The EOP was calculated by dividing the phage titer (PFU/mL) obtained on each susceptible bacterial strain by the phage titer obtained on the original host strain. EOP values were classified as highly productive (≥0.5), medium productive (0.1 ≤ EOP < 0.5), low productive (0.001 < EOP < 0.1), or inefficient (≤ 0.001). All experiments were performed independently in triplicate.

### Phage genome sequencing and analysis

High-titer phage lysates were concentrated using polyethylene glycol (PEG) precipitation. The lysate was combined with sodium chloride (NaCl) and PEG 8000 to final concentrations of 1 M and 10% (wt/vol), respectively. Following incubation overnight at 4°C, the mixture was centrifuged at 7,000 rpm for 30 min at 4°C. The supernatant was discarded, and the resulting pellet was air-dried prior to resuspension in deionized water. To remove contaminating bacterial genetic material, the purified phage preparation was treated with 2 units of DNase I and then incubated at 37°C for 30 min. Subsequently, 0.1 M EDTA was added to achieve a final concentration of 5 mM, and the mixture was incubated at 75°C for 10 min to inactivate the DNase. Phage capsids were then digested by incubating the sample with 10 mg/mL proteinase K and 0.5% sodium dodecyl sulfate (SDS) at 65°C for 30 min. Phage DNA was extracted using the phenol-chloroform-isoamyl alcohol method. Specifically, an equal volume of phenol-chloroform-isoamyl alcohol (25:24:1) was added to the lysed phage solution, and the mixture was vigorously vortexed. The solution was centrifuged at 10,000 rpm for 10 min, and the upper aqueous layer was transferred to a new microcentrifuge tube. This extraction step was repeated as necessary. DNA precipitation was achieved by adding 0.3 volume 3 M sodium acetate (CH_3_COONa) and 1 volume of isopropanol, followed by gentle mixing and incubation at −20°C overnight. The supernatant was discarded, and the pellet was washed with 70% ethanol before centrifugation under the same conditions. After removal of the ethanol, the DNA pellet was air-dried until clear and subsequently dissolved in deionized water. DNA quality was assessed by measuring the absorbance ratio at 260/280 nm, and concentration was determined using a Nanodrop spectrophotometer. The purified DNA was stored at −20°C until further use.

Whole-genome sequencing was conducted utilizing the Illumina MiSeq platform. Following the quality assessment of the FASTQ sequencing reads, *de novo* genome assembly into contigs was executed using SPAdes v3.11.1. Putative open reading frames (ORFs) were predicted and analyzed utilizing the Galaxy platform and PHASTER server, followed by functional annotation via NCBI BLASTp. Intergenomic pairwise similarities among related bacteriophages were quantified using the VIRIDIC tool, and multi-locus comparative genomic analyses were performed with the DiGAlign web server. Finally, phylogenetic relationships were inferred based on the Genome-BLAST Distance Phylogeny (GBDP) method via the VICTOR web service with 100 bootstrap replicates, and the resulting trees were visualized using the Interactive Tree of Life (iTOL) platform.

### Synergy activity of phage-antibiotics combination

To evaluate the efficacy of the phage-antibiotic combinations, five antibiotics with distinct mechanisms of action were used: the cell wall synthesis inhibitor meropenem (MEM), the cell membrane disruptor colistin (CST), the RNA transcription inhibitor rifampin (RIF), the DNA replication inhibitor ciprofloxacin (CIP), and the protein synthesis inhibitor chloramphenicol (CHL). Treatment efficacy was assessed using a 96-well microtiter plate assay. Specifically, 100 µL of bacterial suspension (2.5 × 10⁷ CFU/mL) was inoculated into each well. For the antibiotic-monotherapy groups, each well received 100 µL of TSB, 50 µL of SM buffer, and 50 µL of the respective antibiotic solution (administered at 1 × MIC for MEM and CIP and 0.5 × MIC for CST, RIF, and CHL). For the phage monotherapy groups, 100 µL of TSB, 50 µL of PBS, and 50 µL of the phage suspension were added to achieve a multiplicity of infection (MOI) of 0.01. For the combinatorial treatment groups, treatments comprised 50 µL of the respective antibiotic solution (1 × MIC for MEM and CIP; 0.5 × MIC for CST, RIF, and CHL) and 50 µL of the phage suspension (MOI = 0.01) per well. The microtiter plates were subsequently incubated at 37°C. After 24 and 48 h of incubation, viable bacterial and viral titrations were quantified using the agar dilution plate-counting method. Interaction plots were constructed from bacterial count data obtained after 24 and 48 h of treatment to visualize and qualitatively interpret the interaction between phage alone, antibiotic alone, and phage-antibiotic combination treatments. The interaction was interpreted based on the relationship between the lines in the interaction plots, where non-parallel lines indicated an interaction between phage and antibiotic, whereas parallel lines indicated no interaction (26). The interaction was classified according to the Δlog₁₀ CFU/mL criteria as follows: synergy (Δlog₁₀ CFU/mL ≥2), additive (Δlog₁₀ CFU/mL 1–<2), indifferent (Δlog₁₀ CFU/mL 0–<1), and antagonism (Δlog₁₀ CFU/mL <0). Synergy was defined as a ≥2-log₁₀ CFU/mL reduction in bacterial counts produced by the phage-antibiotic combination compared with the most effective single-agent treatment after 24 and 48 h (17). All experiments were conducted independently in triplicate.

### Effects of phage and antibiotic treatments on bacterial morphology and phage plaque size

#### Bacterial cytological profiling after antibiotics treatment

To assess antibiotic-induced morphological alterations, overnight cultures of the CRAB strain PT061 were diluted 1:100 in fresh medium and incubated at 37°C until reaching the exponential growth phase. The cultures were subsequently treated with specific antibiotics for 1 h at the following concentrations: MEM, CST, and CHL at 5 × MIC; RIF at 1 × MIC; and CIP at 2 × MIC. Following incubation, the treated cells were harvested by centrifugation and stained with the fluorescent dyes FM 4-64 (2 µg/mL) and DAPI (2 µg/mL). After an additional centrifugation step to remove excess dye, 3 µL of the cell suspension was immobilized onto a 1.2% agarose pad prepared with 20% TSB medium. Morphological observations and imaging were then conducted utilizing a ZEISS AxioObserver 7 microscope.

#### Bacterial cytological profiling after phage-antibiotic combination treatment

To evaluate bacterial morphological alterations following combined phage-antibiotic treatment, overnight cultures of CRAB strain PT061 were diluted 1:100 in fresh medium and incubated at 37°C until reaching the exponential growth phase. The cultures were first treated with specific antibiotics for 1 h at the following concentrations: MEM, CST, and CHL at 5 × MIC; RIF at 1 × MIC; and CIP at 2 × MIC. Following initial antibiotic exposure, the cultures were infected with the bacteriophage at a multiplicity of infection (MOI) of 0.01 and incubated at 37°C for an additional 20 min. The treated cells were then harvested via centrifugation and co-stained with the fluorescent dyes FM 4-64 (2 µg/mL) and DAPI (2 µg/mL). After a final centrifugation wash step, 3 µL of the bacterial suspension was immobilized onto a 1.2% agarose pad (prepared in 20% TSB medium). The samples were subsequently visualized utilizing a ZEISS AxioObserver 7 fluorescence microscope.

#### Effect of antibiotics on phage plaque size

To investigate the influence of antibiotics on the plaque dimensions of phiAR008, a DLA assay was performed. Initially, a screening assay was conducted to identify which antibiotics possessed the capacity to increase plaque size. Logarithmic-phase cultures of the CRAB host strain were treated with each antibiotic at a concentration of 10 × MIC. Preliminary observations indicated that MEM and CIP notably induced plaque enlargement. To determine whether this effect was dose-dependent, log-phase CRAB cultures were subsequently exposed to varying concentrations of MEM or CIP (1×, 5×, 10×, and 15 × MIC). After a 24-h incubation at 37°C, phage plaque diameters were measured and recorded. All experiments were performed as three independent biological replicates.

### Effect of antibiotics on phage adsorption and phage production

#### Effect of meropenem and ciprofloxacin on phage adsorption rate

To determine whether the morphological enlargement of bacterial cells induced by MEM or CIP treatment influences phage adsorption kinetics, an adsorption assay was performed. The parental host cells were grown to the logarithmic phase and subsequently treated with either MEM or CIP at 1 × MIC to induce cellular enlargement. Following a 1-h incubation at 37°C, the pretreated cultures were infected with phages at an MOI of 0.01 and maintained at 37°C. To quantify adsorption, 300 µL aliquots were collected at defined time intervals (0, 3, 6, 9, 12, and 15 min) and immediately filtered through a 0.45 µm membrane to remove bacterial cells. The concentration of unadsorbed free phages in the filtrates was subsequently determined via standard enumeration. All experiments were conducted independently in triplicate. The phage adsorption rate constant (k) was calculated using the following equation:


$$k=\frac{2.3}{B\times t}log\left(\frac{P_{0}}{P}\right)$$

where k represents the adsorption rate constant (mL/min), B is the concentration of bacterial cells (cells/mL), t is the time interval (min), $P_{0}$ is the initial phage titer, and P is the concentration of free phages remaining at time t.

#### Effect of meropenem and ciprofloxacin on phage production

To determine whether cellular enlargement induced by MEM or CIP treatment influences phage replication kinetics, a one-step growth curve assay was conducted to evaluate the latent period and burst size. Five milliliters of a logarithmic-phase host culture was treated with either MEM or CIP at 1 × MIC and incubated at 37°C for 1 h. Pretreated cultures were subsequently infected with phage at an MOI of 0.01 and incubated at 37°C for 10 min to facilitate adsorption. Following centrifugation at 4,500 rpm for 5 min, the supernatant containing free virions was collected to quantify the number of unadsorbed phages. This value was subtracted from the initial phage inoculum to accurately estimate the number of infected cells. The bacterial pellet was then resuspended in 6 mL of TSB and incubated at 37°C. Over a 50-min period, aliquots were withdrawn at 10-min intervals and filtered through a 0.45 µm membrane. Phage titers in the filtrates were determined using a spot titer assay via the double-layer agar method. The latent period was defined as the time elapsed from the initiation of infection to the onset of progeny phage release. Burst size was calculated by subtracting the initial unadsorbed phage count from the average maximum phage yield, divided by the total number of infected cells (expressed as PFU/CFU). All experiments were performed independently in triplicate.

In parallel, the quantification of intracellular phage particles post-infection was performed. The CRAB strain PT061 cells were cultivated to the logarithmic phase, treated with MEM or CIP at 1 × MIC, and incubated at 37°C for 1 h prior to infection with phages (MOI = 0.01) at 37°C. Samples were harvested at 5, 10, 15, and 20 min post-infection. Following centrifugation, the infected cells were washed twice with SM buffer to remove extracellular bound and unbound phages. To release intracellular virions, the cells were chemically lysed using chloroform at a final concentration of 10% (vol/vol) and incubated on ice for 1 h. The mixture was subsequently centrifuged, and the aqueous phase was titrated to determine the concentration of intracellular phages per milliliter. Each assay was performed as three independent biological replicates.

#### Phage adsorption on bacterial cell surfaces after treatment with meropenem and ciprofloxacin

To determine whether the morphological enlargement of bacterial cells induced by MEM or CIP treatment influences phage adsorption kinetics, an adsorption assay was performed. The parental host cells were grown to the logarithmic phase and subsequently treated with either MEM or CIP at 1, 2, 5, and 10 × MIC to induce cellular enlargement. Following a 1-h incubation at 37°C, the pretreated cultures were infected with phages at an MOI of 0.0001 and incubated at 37°C. To quantify adsorption, 300 µL aliquots were collected at defined time intervals (6 min) and immediately filtered through a 0.45 µm membrane to remove bacterial cells. The concentration of unadsorbed free phages in the filtrates was subsequently determined via standard enumeration. All experiments were conducted independently in triplicate. The phage adsorption rate constant (k) was calculated using the equation as mentioned above. Bacterial cytological profiling was subsequently visualized using a ZEISS Axio Observer 7 fluorescence microscope, as described above. The correlation (Pearson) between the phage adsorption rate and bacterial surface area was analyzed using GraphPad Prism.

#### Quantification of intracellular phage infection in bacterial cells after treatment with meropenem and ciprofloxacin

The quantification of intracellular phage particles post-infection was performed. The CRAB strain PT061 cells were cultivated to the logarithmic phase, treated with MEM or CIP at 1, 2, 5, and 10 × MIC, and incubated at 37°C for 1 h prior to infection with phages (MOI = 0.0001) at 37°C. Samples were harvested at 6 min post-infection. Following centrifugation, the infected cells were washed twice with SM buffer to remove extracellular bound and unbound phages. To release intracellular virions, the cells were chemically lysed using chloroform at a final concentration of 10% (vol/vol) and incubated on ice for 1 h. The mixture was subsequently centrifuged, and the aqueous phase was titrated to determine the concentration of intracellular phages per infected cell. Each assay was performed as three independent biological replicates. The correlation (Pearson) between the intracellular phage particles and bacterial surface area was analyzed using GraphPad Prism as mentioned above.

#### Phage adsorption on bacterial cell surfaces after treatment with meropenem and ciprofloxacin by transmission electron microscopy

To determine phage adsorption, exponentially growing CRAB PT061 cells were treated with MEM (5 × MIC) or CIP (2 × MIC) at 37°C for 1 h prior to infection with phage phiAR008 at an MOI of 10. Following incubation for 15 min at 37°C to allow phage adsorption, bacterial suspensions were applied onto carbon-coated copper grids and allowed to adsorb for several minutes. The grids were negatively stained with 2% (wt/vol) uranyl acetate for 1 min, followed by rinsing and blotting. The stained grids were then air-dried for 10 min before examination using a Thermo Scientific Talos F200i transmission electron microscope operated at an accelerating voltage of 200 kV.

### Evaluation of the bactericidal activity of phage-antibiotic combinatorial treatments *in vitro and in vivo*

#### Quantification of membrane permeability of phage-antibiotic combination using SYTOX staining by fluorescence microscopy

To assess membrane permeability induced by the combinatorial effects of antibiotics and phiAR008, bacterial cells were subjected to simultaneous phage-antibiotic treatment. The bacterial cultures were co-treated with either MEM or CIP at 1 × MIC alongside phage phiAR008 at an MOI of 0.01, 0.1, or 1.0 and subsequently incubated at 37°C for 20 min. Following this treatment, the cells were harvested via centrifugation and co-stained with the fluorescent dyes SYTOX (1 µg/mL), FM 4-64 (2 µg/mL), and DAPI (1.5 µg/mL). After an additional centrifugation step to remove excess dye, 3 μL of the cell suspension was immobilized onto a 1.2% agarose pad (prepared in 20% TSB medium). The samples were visualized utilizing a ZEISS AxioObserver 7 fluorescence microscope. Cells exhibiting positive SYTOX fluorescence were classified as membrane-permeabilized, and the percentage of permeabilized cells was quantified relative to the total cell count.

#### *In vitro* time-kill assay of phage-antibiotic combination

To evaluate the bactericidal activity of the phage-antibiotic combinations, time-kill assays were performed using CRAB strain PT061 in the logarithmic growth phase. The bacterial cultures were co-treated with MEM or CIP at a concentration of 1 × MIC, alongside phage phiAR008 at an MOI of 0.01, 0.1, or 1.0. All treatment groups were incubated continuously at 37°C. To assess bactericidal kinetics, aliquots were collected at specific time intervals (0, 1, 2, 3, 4, 5, 6, and 24 h post-treatment), and viable bacterial cell counts were quantified. All experiments were conducted independently in triplicate.

#### *In vivo* bacterial activity of phage-antibiotic combination

To evaluate the therapeutic efficacy of phage-antibiotic combinations against bacterial CRAB strains PT061, a *Galleria mellonella* infection model was employed as previously described (48). Larvae were obtained from a local supplier and maintained in the dark for 10 days. Healthy larvae weighing 200–250 mg were randomly selected (*n* = 10 per group), surface-sterilized with 70% ethanol, and kept overnight in 90 mm Petri dishes in the dark prior to experimentation. The CRAB strain PT061 at the logarithmic growth phase was harvested by centrifugation and washed twice with PBS. The larvae were randomly assigned into 10 groups (10 larvae per group) as follows: (i) control, PBS + SM (ii); CRAB strain PT061 (1 × 10^5^ CFU/mL) + SM; (iii) phiAR008 alone (1 × 10^6^ PFU/mL) + PBS; (iv) phiAR008 alone (1 × 10^7^ PFU/mL) + PBS; (v) MEM alone (1 × MIC) + PBS; (vi) CIP alone (1 × MIC) + PBS; (vii) combination treatment at an MOI of 10: MEM (1 × MIC) + phiAR008 (1 × 10^6^ PFU/mL); (viii) combination treatment at an MOI of 100: MEM (1 × MIC) + phiAR008 (1 × 10^7^ PFU/mL); (ix) combination treatment at an MOI of 10: CIP (1 × MIC) + phiAR008 (1 × 10^6^ PFU/mL); and (x) combination treatment at an MOI of 100: CIP (1 × MIC) + phiAR008 (1 × 10^7^ PFU/mL). A 10 µL aliquot of the CRAB strains PT061 suspension was injected into the right posterior proleg of each larva using an insulin syringe (Ultra-Fine II). Larvae were then incubated in the dark in 90 mm plastic Petri dishes for 1 h, after which 10 µL of the designated treatment or combination at the specified concentrations was injected into the left posterior proleg. Larval survival was monitored daily for 5 days, and survival curves were generated using the Kaplan–Meier method. At the end of the experiment, the bacterial loads from the surviving larvae were quantified by plating on MacConkey agar. Each independent experiment was performed in triplicate.

#### Isolation of *in vivo*-derived phage-resistant mutants and determination of minimum inhibitory concentration (MIC)

To isolate phage-resistant bacteria, single colonies were selected from MacConkey agar plates and purified through three successive rounds of subculturing. To confirm the acquisition of phage resistance, aliquots of phage phiAR008 were spotted onto bacterial lawns of each purified isolate. Subsequently, antimicrobial susceptibility profiling evaluating susceptibility to meropenem (MEM), ceftazidime (CFZ), ciprofloxacin (CIP), and chloramphenicol (CHL) was conducted for both the parental CRAB strain PT061 and the phage-resistant isolates using the standard broth microdilution method.

### Statistical analysis

Statistical analyses were performed using GraphPad Prism version 10 (GraphPad Software, San Diego, CA, USA). Group comparisons for CRAB growth following phage-antibiotic combination treatment, phage plaque size under different MEM concentrations, MEM- and CIP-induced effects on phage adsorption and intracellular accumulation, and the antibacterial activities of phage phiAR008 at different MOIs in combination with MEM or CIP in both *in vitro* and *in vivo* experiments were analyzed using one-way analysis of variance (ANOVA), followed by Tukey’s honestly significant difference (HSD) post hoc test. Pearson’s correlation analysis was performed to evaluate the relationships between bacterial surface area and (i) the phage adsorption rate and (ii) the number of intracellular phage particles following antibiotic treatment. Statistical significance was defined as *P* ≤ 0.05. Statistical significance in the figures is indicated as follows: *P* ≤ 0.05 (*), *P* ≤ 0.01 (**), *P* ≤ 0.001 (***), and *P* ≤ 0.0001 (****).

## Data Availability

The genome sequence of phage is available in GenBank under accession number PZ166598.

## SUPPLEMENTAL MATERIAL

Tables S1 and S2, and Fig. S1 to S3 spectrum.01424-26-s0001.pdf The functional annotation of ORFs in phage phiAR008, phage plaque morphology, validation of phage-antibiotic synergy in additional CRAB clinical isolates with different phage infectivity, plaque morphology, and plaque sizes of phage phiAR008 following exposure to MEM, CST, CHL, RIF, and CIP.

ASM does not own the copyrights to Supplemental Material that may be linked to, or accessed through, an article. The authors have granted ASM a non-exclusive, world-wide license to publish the Supplemental Material files. Please contact the corresponding author directly for reuse.
