# Supplementary material for: Antibiotic-induced cellular enlargement drives phage synergy and resensitizes carbapenem-resistant Acinetobacter baumannii
Source: Microbiol Spectr. 2026 Aug 28;14(10):e01424-26. doi: 10.1128/spectrum.01424-26 (PMC13689466; doi:10.1128/spectrum.01424-26)
Supplement: Supplementary file 1 [file spectrum.01424-26-s0001.pdf]

**Table S1** The functional annotation of ORFs in phage phiAR008.

| | CDS | Start | Stop | Size (nt) | Predicted function | NCBI Sequence Hits | Accession number | Per. Ident | E value |
|---|---|---|---|---|---|---|---|---|---|
| 1 | 4 | 2877 | 2494 | 384 | endoribonuclease translational repressor of early genes | Acinetobacter phage AbTZA1 | YP_009882316.1 | 100.00% | 2.00E-91 |
| 2 | 5 | 3443 | 2874 | 570 | clamp loader small subunit | Acinetobacter phage Maestro | QQM13835.1 | 98.94% | 7.00E-139 |
| 3 | 7 | 5224 | 4535 | 690 | DNA polymerase sliding clamp | Acinetobacter phage AbTZA1 | YP_009882313.1 | 100.00% | 7.00E-167 |
| 4 | 8 | 5636 | 5262 | 375 | RNA polymerase binding | Acinetobacter phage AbTZA1 | YP_009882312.1 | 100.00% | 1.00E-88 |
| 5 | 12 | 8388 | 7798 | 591 | phosphoesterase | Acinetobacter phage vB_ApiM_fHyAci03 | YP_009880876.1 | 69.95% | 3.00E-108 |
| 6 | 16 | 10522 | 9980 | 543 | sigma factor | Acinetobacter phage vB_AbaM_DLP | XEO41493.1 | 94.41% | 5.00E-126 |
| 7 | 18 | 11305 | 11024 | 282 | nucleotide reductase | Acinetobacter phage AC4 | UQS93691.1 | 87.10% | 1.00E-55 |
| 8 | 20 | 12028 | 11546 | 483 | endonuclease VII | Acinetobacter phage AbTZA1 | YP_009882300.1 | 99.38% | 1.00E-115 |
| 9 | 37 | 21017 | 20559 | 459 | membrane protein | Acinetobacter phage vB_AbaM_PhT2-v2 | CAL1778000.1 | 88.82% | 8.00E-92 |
| 10 | 38 | 21289 | 21023 | 267 | DksA-like zinc-finger protein | Acinetobacter phage AbTZA1 | YP_009882282.1 | 98.86% | 1.00E-61 |
| 11 | 39 | 21626 | 21324 | 303 | lysis inhibition | Acinetobacter phage AbTZA1 | YP_009882281.1 | 100.00% | 1.00E-69 |
| 12 | 42 | 22670 | 22344 | 327 | Vs valyl-tRNA synthetase modifier | Acinetobacter phage AB2I2 | XJP41221.1 | 81.48% | 4.00E-62 |
| 13 | 44 | 23761 | 23330 | 432 | homing endonuclease | Acinetobacter phage AbTZA1 | YP_009882276.1 | 100.00% | 1.00E-103 |
| 14 | 57 | 27822 | 27256 | 567 | tail fiber protein | Acinetobacter phage KARL-1 | YP_009881557.1 | 78.19% | 1.00E-107 |
| 15 | 58 | 28132 | 27896 | 237 | peptide chain release factor | Acinetobacter phage Minot | QQO96345.1 | 96.15% | 2.00E-50 |
| 16 | 64 | 30243 | 29785 | 459 | RNA ligase | Acinetobacter phage AbTZA1 | YP_009882264.1 | 100.00% | 2.00E-111 |
| 17 | 65 | 30485 | 30243 | 243 | tail fiber chaperone | Acinetobacter phage vB_ApiM_fHyAci03 | YP_009880921.1 | 86.15% | 1.00E-33 |
| 18 | 66 | 31206 | 30472 | 735 | deoxynucleoside monophosphate kinase | Acinetobacter phage vB_AbaM_Lazarus | YP_009886922.1 | 62.18% | 1.00E-100 |
| 19 | 67 | 31772 | 31203 | 570 | tail completion and sheath stabilizer | Acinetobacter phage Maestro | QQM13894.1 | 99.47% | 2.00E-138 |
| 20 | 69 | 33279 | 32830 | 450 | head closure | Acinetobacter phage AbTZA1 | YP_009882259.1 | 100.00% | 1.00E-108 |
| 21 | 70 | 33341 | 33934 | 594 | baseplate wedge subunit | Acinetobacter phage vB_AbaM_Apostate | YP_009886429.1 | 92.35% | 3.00E-136 |
| 22 | 79 | 45232 | 45891 | 660 | baseplate wedge subunit and tail pin protein | Acinetobacter phage vB_AbaM_DLP3 | XEO41481.1 | 82.19% | 3.00E-134 |
| 23 | 85 | 51905 | 52390 | 486 | terminase small subunit | Acinetobacter phage AbTZA1 | YP_009882243.1 | 96.27% | 3.00E-114 |
| 24 | 90 | 57288 | 57779 | 492 | tail protein | Acinetobacter phage AM101 | YP_009889807.1 | 97.55% | 9.00E-119 |
| 25 | 92 | 59394 | 59609 | 216 | prohead core protein | Acinetobacter phage Maestro | QQM13919.1 | 97.18% | 2.00E-40 |
| 26 | 93 | 59609 | 60037 | 429 | head scaffolding protein | Acinetobacter phage vB_ApiM_fHyAci03 | YP_009880948.1 | 90.14% | 3.00E-88 |

**Table S1** The functional annotation of ORFs in phage phiAR008 (cont.).

| | CDS | Start | Stop | Size (nt) | Predicted function | NCBI Sequence Hits | Accession number | Per. Ident | E value |
|---|---|---|---|---|---|---|---|---|---|
| 27 | 94 | 60034 | 60681 | 648 | head maturation protease | Acinetobacter phage AbTZA1 | YP_009882234.1 | 100.00% | 2.00E-157 |
| 28 | 99 | 68366 | 67644 | 723 | minor head protein inhibitor of protease | Acinetobacter phage vB_AbaM_PhT2-v2 | CAL1776984.1 | 99.17% | 3.00E-176 |
| 29 | 107 | 72609 | 72211 | 399 | baseplate wedge subunit | Acinetobacter phage AbTZA1 | YP_009882221.1 | 100.00% | 3.00E-94 |
| 30 | 108 | 73253 | 72609 | 645 | baseplate hub subunit | Acinetobacter phage Maestro | QQM13937.1 | 96.73% | 7.00E-153 |
| 31 | 111 | 75658 | 76209 | 552 | baseplate hub distal subunit | Acinetobacter phage AbTZA1 | YP_009882217.1 | 98.91% | 2.00E-132 |
| 32 | 122 | 85856 | 85614 | 243 | lysis inhibition; accessory protein | Acinetobacter phage vB_AbaM_PhT2 | YP_009887144.1 | 98.75% | 1.00E-53 |
| 33 | 123 | 86264 | 85938 | 327 | head morphogenesis | Acinetobacter phage AbTZA1 | YP_009882204.1 | 99.07% | 1.00E-73 |
| 34 | 125 | 87156 | 86605 | 552 | dCMP deaminase | Acinetobacter phage AbTZA1 | YP_009882202.1 | 99.45% | 2.00E-135 |
| 35 | 127 | 88049 | 87519 | 531 | MutT/NUDIX hydrolase | Acinetobacter phage vB_AbaM_PhT2 | YP_009887139.1 | 99.43% | 2.00E-129 |
| 36 | 132 | 90256 | 89771 | 486 | inhibitor of host transcription | Tegunavirus sp. BRC001 | CAL1777413.1 | 96.89% | 1.00E-114 |
| 37 | 142 | 99362 | 98820 | 543 | dihydrofolate reductase | Acinetobacter phage vB_AbaM_PhT2-v2 | CAL1777261.1 | 98.33% | 2.00E-133 |
| 38 | 148 | 102712 | 102062 | 651 | DNA helicase loader | Acinetobacter phage LemonAid | WPH64912.1 | 91.67% | 3.00E-145 |
| 39 | 149 | 103001 | 102705 | 297 | late promoter transcriptional regulator [ | Tegunavirus sp. BRC001 | CAL1777316.1 | 100.00% | 4.00E-67 |
| 40 | 150 | 103248 | 102982 | 267 | transcriptional regulator | Acinetobacter phage AbTZA1 | YP_009882178.1 | 100.00% | 4.00E-59 |
| 41 | 155 | 109954 | 110658 | 705 | hinge connector of long tail fiber protein distal connector | Acinetobacter phage vB_AbaM_PhT2 | YP_009887111.1 | 100.00% | 4.00E-171 |
| 42 | 159 | 116156 | 115881 | 276 | anti-sigma 70 protein | Acinetobacter phage Octan | QKE55943.1 | 95.60% | 2.00E-58 |
| 43 | 163 | 119068 | 118610 | 459 | Ndd-like nucleoid disruption protein | Acinetobacter phage vB_AbaM_Konradin | YP_009885431.1 | 90.13% | 6.00E-100 |
| 44 | 164 | 119732 | 119130 | 603 | DenB-like DNA endonuclease IV | Acinetobacter phage AbTZA1 | YP_009882164.1 | 99.00% | 2.00E-149 |
| 45 | 176 | 128784 | 128221 | 564 | endolysin | Acinetobacter phage AbTZA1 | YP_009882155.1 | 100.00% | 3.00E-136 |
| 46 | 178 | 129554 | 129312 | 243 | cef modifier of supressor tRNAs | Acinetobacter phage vB_AbaM_PhT2 | YP_009887090.1 | 93.75% | 3.00E-50 |
| 47 | 180 | 130674 | 130015 | 660 | exonuclease | Acinetobacter phage Maestro | QQM13748.1 | 99.54% | 1.00E-163 |
| 48 | 212 | 144130 | 143612 | 519 | dUTPase | Acinetobacter phage vB_AbaM_PhT2 | YP_009887055.1 | 100.00% | 7.00E-127 |
| 49 | 239 | 156627 | 156316 | 312 | head vertex assembly chaperone | Acinetobacter phage AB-Navy71 | UQS94149.1 | 95.15% | 1.00E-67 |
| 50 | 242 | 159477 | 158935 | 543 | thymidylate kinase | Tegunavirus sp. BRC001 | CAL1778043.1 | 94.44% | 2.00E-123 |
| 51 | 243 | 160409 | 159465 | 945 | thymidylate synthase | Acinetobacter phage AM101 | YP_009889705.1 | 91.42% | 3.00E-163 |
| 52 | 247 | 163860 | 163228 | 633 | arabinose 5-phosphate isomerase | Acinetobacter phage Acj61 | YP_004009701.1 | 86.67% | 9.00E-138 |

**Table S2** List of annotated proteins from tRNA of phiAR008.

| | tRNA | Start | Stop | Size (nt) | Product |
|---|---|---|---|---|---|
| 1 | 49 | 25557 | 25486 | 72 | tRNA-Trp(cca) |
| 2 | 51 | 25963 | 25875 | 89 | tRNA-Ser(gct) |
| 3 | 53 | 26363 | 26287 | 77 | tRNA-Pro(tgg) |
| 4 | 54 | 26577 | 26483 | 95 | tRNA-Ser(tga) |
| 5 | 55 | 26858 | 26782 | 77 | tRNA-Arg(tct) |
| 6 | 56 | 26943 | 26872 | 72 | tRNA-Thr(tgt) |
| 7 | 59 | 28228 | 28152 | 77 | tRNA-Met(cat) |
| 8 | 61 | 29039 | 28965 | 75 | tRNA-Pyl(cta) |

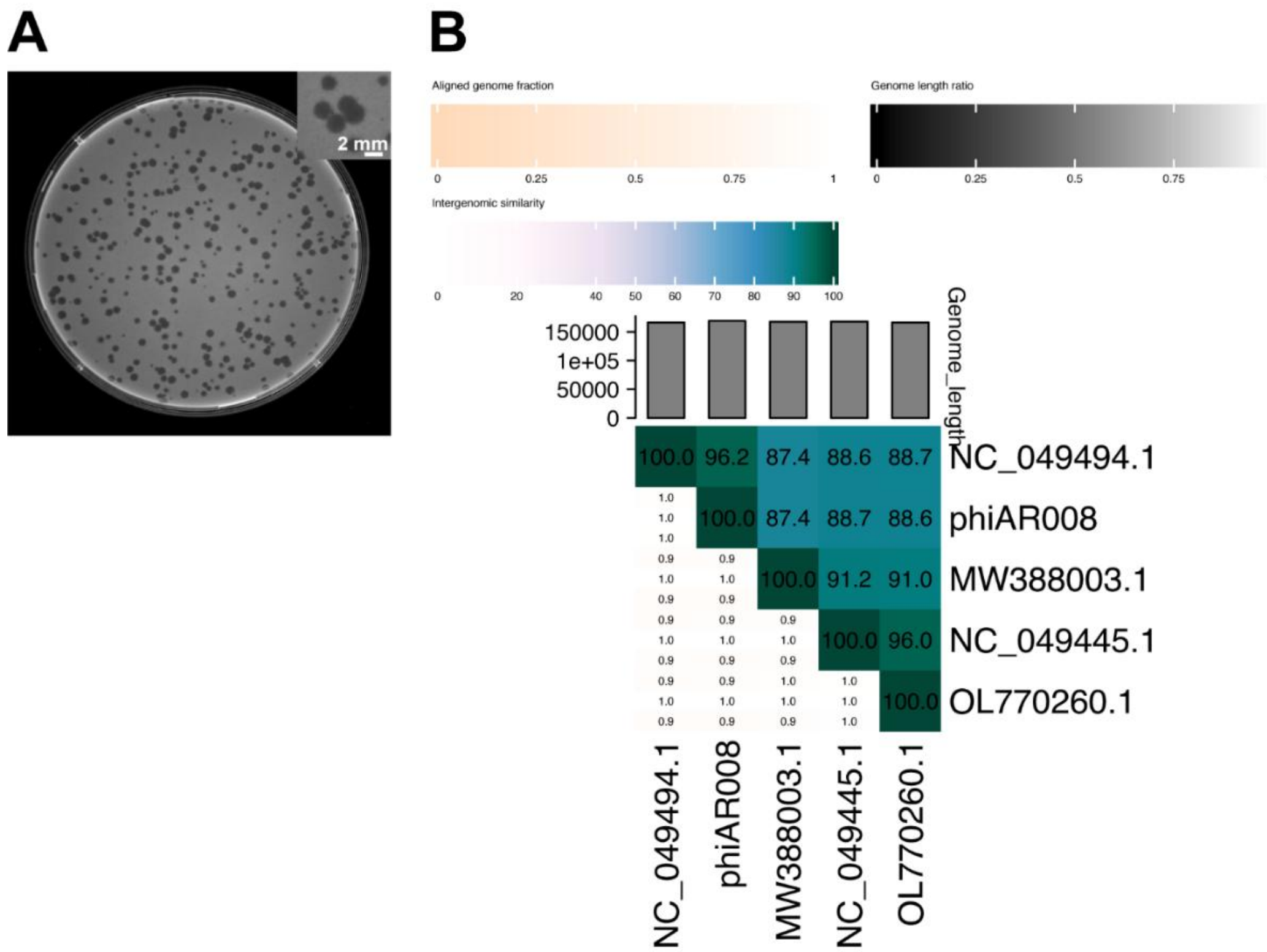


**Fig. S1 (A)** Phage plaque morphology was observed on TSB supplemented with 0.35% agar and incubated at 37°C for 24 h. **(B)** A VIRIDIC heatmap showed the intergenomic similarities among phages phiAR008 and related phages. The values indicate the percentage similarity between genome pairs.

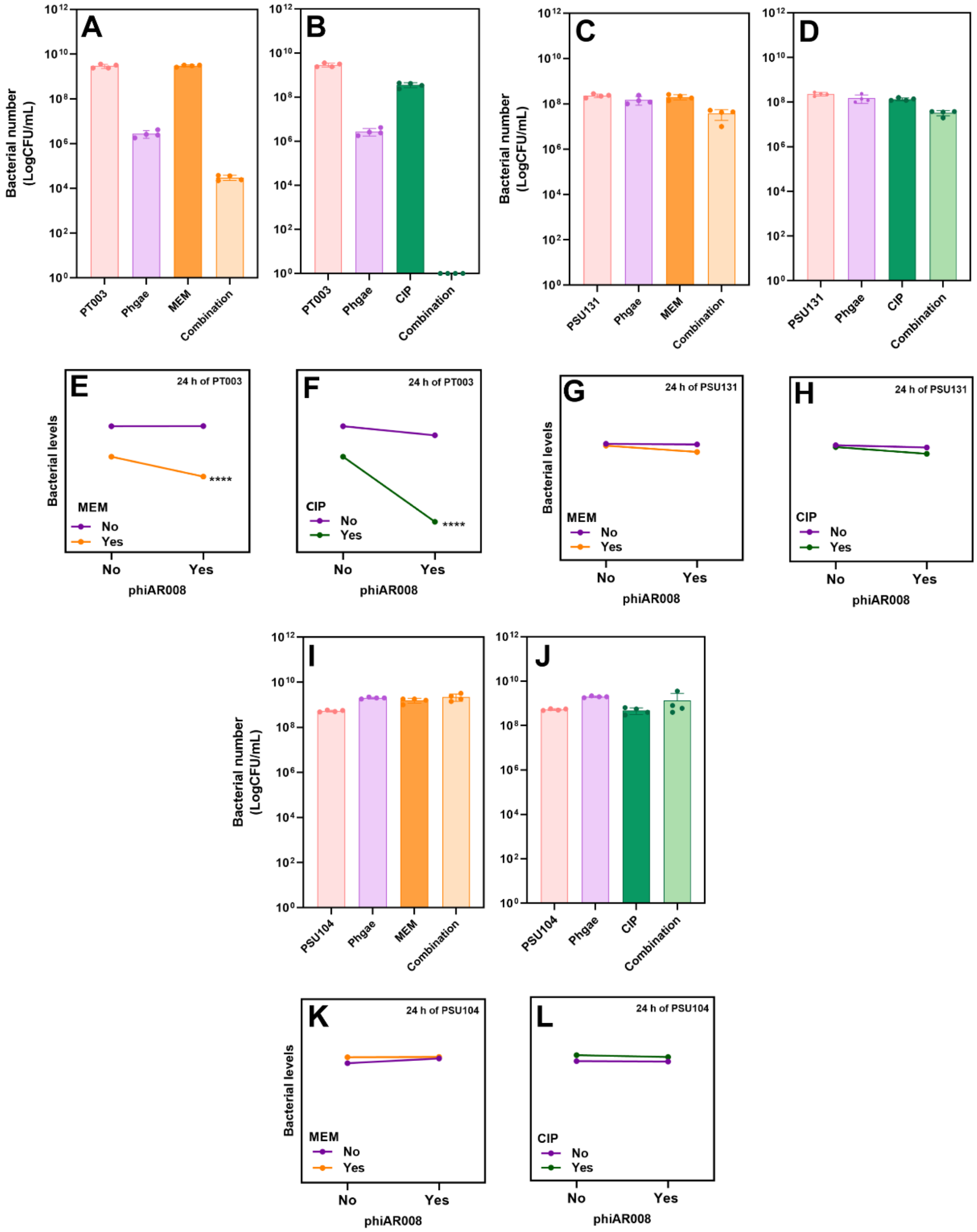


**Fig. S2** Validation of phage-antibiotic synergy in additional CRAB clinical isolates with different phage infectivity. **(A–B)** Bacterial counts of strain PT003 after 24 h treatment with phage phiAR008 alone, antibiotic alone (MEM or CIP), or the combination treatment. The combination treatment resulted in significantly greater bacterial killing than either treatment alone. **(C–D)** Bacterial counts of strain PSU131 after treatment with phage phiAR008, MEM or CIP alone, and their combinations. No obvious synergistic effect was observed in this strain. **(E–H)** Interaction plots showing the effects of phage phiAR008 and MEM or CIP on bacterial levels in PT003 and PSU131 after 24 h. Significant interaction between phage and antibiotics was observed only in PT003, indicating phage-antibiotic synergy. **(I–J)** Bacterial counts of strain PSU104 following treatment with phage phiAR008, MEM or CIP alone, and their combinations. Neither phage treatment nor phage-antibiotic combinations reduced bacterial growth. **(K–L)** Interaction

plots showing the effects of phage phiAR008 and MEM or CIP on bacterial levels in PSU104 after 24 h. No significant interaction between phage and antibiotics was detected. Data are presented as the mean ± standard deviation of at least three independent biological replicates. Statistical analysis for panels **E-F** was performed using one-way ANOVA followed by Tukey's HSD test (*$p \leq 0.05$, **$p \leq 0.01$, ***$p \leq 0.001$, and ****$p \leq 0.0001$).

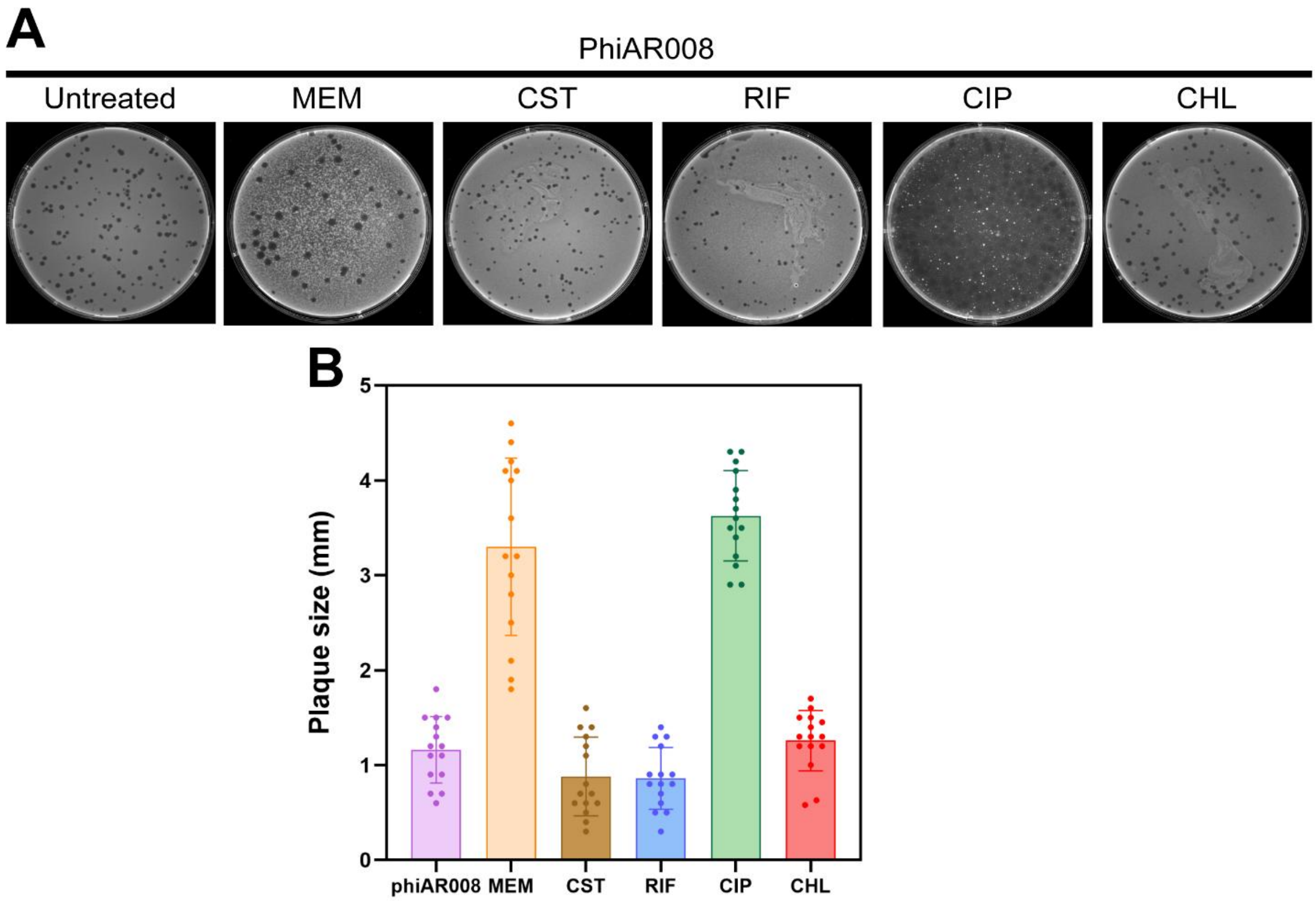


**Fig. S3 (A)** Plaque morphology and **(B)** plaque sizes of phage phiAR008 following exposure to MEM, CST, CHL, RIF, and CIP at a concentration of 10× MIC
